# Exploring the Potential of Zein Nanoparticles in Personalised Cancer Therapy, Highlighting Their Various Methodologies, Applications and Challenges

**DOI:** 10.1111/jcmm.70752

**Published:** 2025-08-12

**Authors:** Hanan M. Alharbi, Taha Alqahtani, Nada A. Alqalawi, Shayma A. Alsayegh, Basmah A. Almaghrabi, Subham Sarkar, Daniel Ejim Uti, Bikram Dhara

**Affiliations:** ^1^ Department of Pharmaceutical Sciences, Faculty of Pharmacy Umm Al‐Qura University Makkah Saudi Arabia; ^2^ Department of Pharmacology, College of Pharmacy King Khalid University Abha Saudi Arabia; ^3^ Postgraduate and Research Department of Biotechnology St. Xavier's College (Autonomous) Kolkata India; ^4^ Department of Research Publications Kampala International University Kampala Uganda; ^5^ Department of Biochemistry, Faculty of Basic Medical Sciences, College of Medicine Federal University of Health Sciences Otukpo Benue State Nigeria; ^6^ Center for Global Health Research, Saveetha Medical College and Hospital Saveetha Institute of Medical and Technical Sciences Chennai India

**Keywords:** advanced drug delivery mechanisms, cancer therapy, oral bioavailability, precision medicine, zein nanoparticles

## Abstract

Zein, a corn‐derived prolamine protein, has become a powerful ally in the fight against cancer, particularly non‐small cell lung cancer (NSCLC.) Its unique attributes, enriched by modifiable hydroxyl and amino groups, have led to the development of advanced functionalised drug delivery systems. Innovative techniques like chemical crosslinking, desolvation, dispersion and micromixing have led to the creation of zein‐based nanoparticles, revolutionising cancer therapy. Central to this examination is the remarkable ability of zein NPs to enhance drug stability, optimise oral bioavailability and improve targeted drug delivery, specifically tailored to combat NSCLC. This represents not just a technological breakthrough but a paradigm shift, ushering in a new era of precise, personalised and effective cancer treatment. Zein, a hydrophobic nanoparticle, is a promising drug for cancer treatment. However, its journey to the clinic is challenging due to its hydrophobic nature and the need for advanced evaluative platforms. This review emphasises the need for rigorous research to align zein's potential with real‐world applications. It offers a synthesis of methodologies, applications, and obstacles, aiming to see zein nanoparticles as a central element in cancer therapy innovations. The review encourages researchers, clinicians and industry professionals to embrace the potential of zein and promote the convergence of laboratory innovation and clinical application.

## Graphical abstract

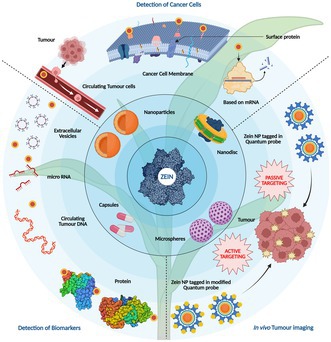

## 1 Introduction

The first two decades of the 21st century stand as a testament to unprecedented medical and technological advancements. Yet, cancer remains a formidable adversary, posing an unrelenting challenge that continually escapes definitive solutions. According to the global statistics for 2020, approximately 19.3 million new cancer cases were diagnosed, resulting in an alarming 10 million fatalities, a trend that is only projected to escalate. Forecasts for 2040 predict a 47% increase in global cancer incidents, reaching an unprecedented 28.4 million cases [1]. Lung cancer stands out in this grim panorama, with 1.8 million annual diagnoses and 1.6 million fatalities, a figure that underscores the critical need for innovative therapeutic strategies [2]. Historically, the primary modalities for cancer treatment have been chemotherapy, surgery and radiotherapy. While these methods have indeed been instrumental in managing and treating various forms of cancer, they present significant limitations. Chemotherapy, for example, is known for its systemic and indiscriminate action, often leading to severe side effects that affect both malignant and normal cells alike [2]. The necessity for more targeted, efficient, and patient‐friendly solutions has never been more pronounced. The intersection of nanomedicine and the versatile protein zein offers a promising pathway in this direction. Zein, a corn‐derived protein first identified in 1821 [3], has seen a resurgence in medical interest. It exhibits a unique hydrophobic nature and a distinctive amino acid profile, allowing for the encapsulation of hydrophobic agents and modulation of drug release over extended periods [4]. It is Generally Regarded as Safe (GRAS) status further amplifies its potential for human applications [5].

Beyond its biocompatibility, zein's versatility extends to its ability to self‐organise into nanoparticles, a feature that establishes its relevance in designing tailor‐made drug delivery mechanisms. Applications have already been found across diverse medical fields including nutrition [6], tissue regeneration [7] and a broad spectrum of biomedical innovations [8]. In the pharmaceutical nanotechnology arena, zein's capabilities are being harnessed to develop state‐of‐the‐art drug delivery systems that target specific cells while minimising collateral damage to healthy tissue [9, 10]. The recent advances in drug delivery owe much to the innovative utilisation of nanotechnology. Traditional challenges such as a lack of targeted delivery and controlled release are being overcome through nanoparticles, which offer benefits like improved drug solubility, shielded therapeutic components and regulated release [11, 12]. Nanoparticles are also being engineered to enhance circulation and targeting, with specific attributes like size, shape and surface topologies determining their effectiveness [10]. The delicate balance of size in the 10–100 nm range is particularly significant in ensuring both effectiveness and safety [11, 12], with hydrophilic coatings further augmenting drug circulation and retention [13, 14, 15].

Current methods for synthesising nanoparticles often lack standardisation, leading to variations in size, shape and properties between different batches and studies. Research is needed to develop reliable and reproducible protocols for synthesis. While various techniques are used for characterisation, a comprehensive understanding of the nanoparticle's surface chemistry, stability in different environments and long‐term behaviour is still lacking. More in‐depth studies using advanced characterisation methods are needed. A thorough investigation of the potential toxicity of specific nanoparticles on different cell types and in vivo models is crucial. Understanding their interactions with biological systems at a molecular level is essential for safe applications. The mechanisms of cellular uptake, biodistribution, and elimination of nanoparticles in living organisms are not fully understood. Research in this area is vital for optimising their delivery and minimising potential side effects. Developing strategies to functionalise nanoparticles for targeted delivery of therapeutic agents to specific cells or tissues remains a significant challenge. Research focusing on surface modification and ligand conjugation is needed. Many current synthesis methods are not easily scalable for industrial production. Research into cost‐effective and sustainable large‐scale production methods is crucial for the widespread application of the nanoparticles. Conventional nanoparticle synthesis often involves hazardous chemicals, high energy consumption, and the generation of toxic byproducts. Plant‐based synthesis, utilising natural reducing and stabilising agents found in plant extracts, offers a greener, more environmentally friendly approach, addressing the critical need for sustainable nanotechnology. Plant extracts (e.g., from *Zea mays* ) are generally inexpensive and readily available, making plant‐based nanoparticle synthesis potentially more cost‐effective for large‐scale production compared to methods requiring specialised equipment and expensive chemicals. Plant‐mediated synthesis often involves simpler processes, reducing the complexity and time associated with nanoparticle fabrication (e.g., antisolvent precipitation/nanoprecipitation, spray drying, liquid–liquid dispersion or supercritical fluid technology). The inherent biocompatibility of zein nanoparticles makes them promising candidates for drug delivery, bioimaging and tissue engineering, due to their natural hydrophobicity, edibility, biodegradability and biocompatibility, potentially overcoming issues of toxicity and immunogenicity associated with other nanomaterials. Zein nanoparticles can be functionalised with specific ligands or antibodies for targeted delivery of therapeutic agents to diseased cells or tissues, improving treatment efficacy and reducing side effects [16, 17, 18, 19]. *Zea mays* possesses inherent antimicrobial and antioxidant properties. Zein nanoparticles can retain or enhance these properties, offering novel solutions to combat drug‐resistant pathogens and oxidative stress‐related diseases. Zein possesses remarkable advantages over other commonly used nano‐therapeutics and drug delivery systems, making it a choice for further research by studying its efficiency in different cancer cell lines (Table 1).

**TABLE 1:** A comparative analysis of biocompatibility, efficiency, stability, storage, and manufacturing and scalability of zein and other commonly used materials for developing nanoscale drug delivery systems.

Material	Origin and biocompatibility	Drug loading and release	Stability and storage	Targeting and cellular uptake	Manufacturing and scalability
Zein	Plant‐derived (corn); GRAS by FDA; excellent biocompatibility and biodegradability	Ideal for hydrophobic drugs; modifiable for hydrophilic drugs; sustained release	Stable under dry conditions; sensitive to humidity and extreme pH	Modifiable with targeting ligands; positive charge enhances uptake	Simple, scalable methods (e.g., antisolvent precipitation); cost‐effective
PLGA	Synthetic; FDA‐approved; biocompatible and biodegradable; produces acidic byproducts	Encapsulates both hydrophobic and hydrophilic drugs; tunable release via composition	Hydrolyzes with moisture; affects stability	Often modified to improve targeting and uptake	Well‐established, scalable methods; organic solvents can be a concern
PCL	Synthetic polyester; good biocompatibility; very slow degradation	Suitable for hydrophobic drugs; prolonged release	More stable than PLGA; slow degradation	Surface functionalization enhances uptake	Similar methods as PLGA; high viscosity may limit scalability
Albumin	Natural protein (bovine/human); excellent biocompatibility; potential immunogenicity	Binds both hydrophilic and hydrophobic drugs; enzymatic/pH‐triggered release	Prone to denaturation (temp, pH, shear); requires careful storage	Conjugation with ligands possible; interacts with albumin‐binding receptors	Scalable de‐solvation methods; source and purification affect cost/reproducibility
Casein	Milk protein; good biocompatibility and biodegradability; interacts with calcium ions	Encapsulates both drug types; pH‐sensitive release	Stable; enzymatic degradation can affect	Modifiable for targeting; calcium interaction good for bone targeting	Achievable via de‐solvation/emulsification; scalable
Gelatin	Derived from collagen; good biocompatibility, cell adhesion	Loads both hydrophilic and hydrophobic drugs; release controlled by degradation/crosslinking	Susceptible to degradation by moisture/temp; crosslinking over time	Modifiable for targeting; natural cell adhesion enhances uptake	Coacervation/Emulsification; scalable
Liposomes	Lipid‐based vesicles; biocompatible and biodegradable	Hydrophilic drugs in core; amphiphilic in bilayer; pH, enzyme, temp‐triggered release	Prone to aggregation, fusion, leakage; needs special storage (e.g., lyophilization)	Ligand modification common for targeting (e.g., folate, antibodies)	Complex, multi‐step processes; challenging large‐scale production
EEVs	Naturally secreted by cells; excellent biocompatibility & targeting potential	Encapsulate proteins, nucleic acids, small molecules; complex release mechanisms	Stability depends on source/purification; often requires low temp or lyophilization	Inherent targeting from cell origin; can be engineered for specificity	Requires cell culture & complex purification; major scalability challenge

Abbreviations: EEV, engineered extracellular vesicles; PCL, polycaprolactone; PLGA, poly—(lactic‐co‐glycolic acid).

Although the creation of zein nanoparticles faces certain challenges, such as insolubility in aqueous environments, innovative solutions are emerging. These include the utilisation of polymer blends, core or shell‐based coatings and cross‐linked zein nanoparticles [20, 21, 22, 23, 24, 25, 26, 27, 28]. Particularly in leukaemia, a complex haematological malignancy, zein‐based nanoparticles have shown promising results. They have enabled targeted delivery of chemotherapeutics, minimised systemic exposure and potentially reduced side effects [11, 12]. This preferential accumulation in leukaemia cells over normal cells is emblematic of the targeted approach that characterises zein's potential in cancer management [13]. While the clinical translation of zein‐based nanoparticles for cancer therapy is still in its relatively early stages, preclinical research has shown promising success in treating various types of cancer in in vitro and in vivo studies. There are also several other gaps which need to addressed, such stability and long‐term storage of drug loaded zein nanoparticles, control over self‐assembly, achieving high batch‐to‐batch consistency, drug release in complex tumour microenvironments, biodistribution and pharmacokinetics in complex systems, specificity and effectiveness in heterogenous tumours, scalability, and regulatory hurdles. To address them, proper research ideas should be encouraged and then rigorously investigated. This review will take an in‐depth and comprehensive look at the synthesis, structure, characterisation and ligand‐based modification of zein nanoparticles, especially in the context of cancer therapeutics. In addition to exploring the role of zein nanoparticles in overcoming cancer drug resistance, it will emphasise on the innovative and groundbreaking prospects that these nanoparticles present in the broader therapeutic landscape, such as multidimensional personalization strategies with patient‐specific ligands and tumour microenvironment tailoring, synergism of multiple modifications, bridging the gap from OMICS to nanomedicine formulation and functionalization and optimization of personalised design using artificial intelligence algorithms and models.

## 2 Comprehensive Overview of Zein Nanoparticles: Structural Characteristics, Morphological Properties and Functional Utility Across Industries

Zein nanoparticles (ZNP) have demonstrated significant potential in overcoming limitations associated with conventional drug delivery systems, such as poor drug solubility, rapid degradation, non‐specific targeting and systemic toxicity (Table 2). Numerous studies have demonstrated the ability of ZNPs to significantly enhance the oral bioavailability of hydrophobic drugs. (a) Scientists have encapsulated cyclosporine A, a poorly water‐soluble immunosuppressant, in zein nanoparticles and observed a significant increase in its oral bioavailability in rats compared to the unformulated drug. The ZNPs protected the drug from enzymatic degradation in the gastrointestinal tract and facilitated its absorption. The hydrophobic core of ZNPs provides a favourable environment for the solubilisation and encapsulation of lipophilic drugs. The nanoparticle matrix can also protect the drug from the harsh acidic environment of the stomach and enzymatic breakdown in the intestines. Furthermore, ZNPs can interact with the intestinal mucosa, potentially enhancing drug permeation through mechanisms like increased residence time and interaction with uptake transporters. Studies have also shown improved oral bioavailability of curcumin, resveratrol and artemether when formulated as ZNPs. (b) ZNPs can be engineered to provide sustained and controlled release of encapsulated drugs. Studies demonstrated the sustained release of a model protein from zein microspheres, highlighting the potential of zein as a matrix for controlled drug delivery. Nanoparticle formulations offer even greater control due to their higher surface area to volume ratio. The dense protein matrix of zein can slow down the diffusion of encapsulated drugs, leading to a prolonged release profile. The release rate can be further tailored by modifying the ZNP size, morphology and surface properties, as well as by incorporating other polymers or additives into the formulation. This controlled release can maintain therapeutic drug concentrations for extended periods, reducing dosing frequency and improving patient compliance. Sustained release of insulin, anticancer drugs like paclitaxel and anti‐inflammatory agents has been achieved using ZNP‐based formulations. (c) Surface modification of ZNPs with targeting ligands allows for selective delivery of drugs to specific cells or tissues, minimising off‐target effects. Conjugated folic acid, a ligand overexpressed on many cancer cells, to zein nanoparticles loaded with doxorubicin. The folate‐conjugated ZNPs exhibited enhanced uptake and cytotoxicity in folate receptor‐positive cancer cells compared to non‐targeted ZNPs. Targeting ligands, such as antibodies, peptides, aptamers or small molecules, can be chemically or physically attached to the surface of ZNPs. These ligands specifically bind to receptors overexpressed on target cells, leading to enhanced internalisation of the drug‐loaded nanoparticles via receptor‐mediated endocytosis. This targeted approach can significantly improve therapeutic efficacy while reducing systemic toxicity. ZNPs have been targeted to tumour vasculature using RGD peptides, to the liver using lactobionic acid, and to inflammatory sites using specific antibodies. (d) ZNPs excel in delivering lipophilic drugs due to their hydrophobic core. Established reports consistently demonstrate improved solubility, stability, and bioavailability of such drugs. While zein is primarily hydrophobic, surface modification and formulation strategies can enable the delivery of hydrophilic molecules. Scientists developed positively charged zein nanoparticles modified with chitosan to encapsulate and deliver plasmid DNA. The electrostatic interaction between the negatively charged DNA and the positively charged nanoparticles facilitated efficient loading and transfection. ZNPs can protect fragile protein and peptide drugs from enzymatic degradation and enhance their stability. Insulin encapsulated in zein nanoparticles coated with Eudragit L100‐55 for oral delivery demonstrated protection in the acidic stomach and release in the intestinal pH. ZNPs have been explored for the delivery of siRNA and other nucleic acid‐based therapeutics for gene therapy. Surface modification with cationic polymers is often employed to facilitate interaction with negatively charged nucleic acids and cellular uptake.

**TABLE 2:** Different therapeutic applications of zein nanoparticle.

Therapeutic application	Established reports	Elaboration
Enhanced oral bioavailability of poorly water‐soluble drugs	Protected drug from enzymatic degradation and aided absorption.	Hydrophobic core of ZNPs solubilizes and encapsulates lipophilic drugs. Protection from gastric acid and enzymes. Interacts with intestinal mucosa to enhance permeation.
Controlled and sustained drug release	Nanoparticles offer better control due to high surface area.	Dense zein matrix slows drug diffusion. Release can be tailored by particle size, surface properties and additives. Maintains therapeutic levels longer, reducing dosing frequency.
Targeted drug delivery to specific tissues and cells	Folate‐conjugated ZNPs loaded with doxorubicin showed selective uptake in cancer cells.	Ligands like antibodies, peptides, and small molecules bind to overexpressed receptors. Facilitates receptor‐mediated endocytosis. Enhances efficacy and reduces side effects.
Delivery of diverse therapeutic agents	Hydrophobic drugs: Strongly supported for lipophilic compounds. Hydrophilic drugs: Chitosan‐modified ZNPs for plasmid DNA. Proteins/Peptides: Insulin using ZNPs coated with Eudragit. Nucleic acids: ZNPs used for siRNA and gene delivery.	ZNPs' hydrophobic core supports lipophilic drug delivery. Surface modifications (e.g., chitosan) enable loading of hydrophilic compounds. Coating strategies improve stability and pH‐triggered release for proteins/peptides. Cationic modifications aid nucleic acid delivery.

Application area	Established reports	Elaboration
Fabrication of scaffolds with tunable properties	Zein can be processed into nanofibers, microparticles, and films for varied tissue applications.	Electrospun fibres mimic ECM; microparticles work as injectables/building blocks; films serve as membranes or substrates. Tailoring scaffold structure helps guide regeneration.
enhanced cell adhesion, proliferation and differentiation	HMSCs adhered and proliferated on electrospun zein scaffolds.	Zein's hydrophobicity and amino acid content influence cell interaction. Surface biofunctionalization (e.g., RGD) and porosity support adhesion and growth.
Delivery of growth factors within scaffolds	BMP‐2 loaded zein microspheres in porous scaffold enhanced osteogenic differentiation.	ZNPs protect and gradually release growth factors, aiding localised regeneration through improved cell activity and matrix formation.
Delivery of Contrast Agents for Medical Imaging	SPIONs encapsulated in ZNPs used as MRI contrast agents; zein shell improved biocompatibility and stability.	Encapsulation increases stability, circulation time, and target‐site accumulation. Surface ligands enhance imaging specificity.
Fluorescent labeling for cell tracking and diagnostics	Fluorescently labelled ZNPs tracked in cancer cells, showing uptake and intracellular movement.	Used for studying nanoparticle uptake and intracellular behaviour; applicable in diagnostics and tracking therapeutic outcomes.
Cosmetics and cosmeceuticals		ZNPs improve penetration and control release of active ingredients. Their biocompatibility suits cosmetic applications.
Antimicrobial applications		Zein has intrinsic antimicrobial traits. ZNPs deliver antimicrobial agents locally and are used in device coatings to reduce infection risks.

Zein can be processed into nanofibers via electrospinning, microparticles via emulsification and solvent evaporation, and films via casting. These different forms provide scaffolds with varying pore sizes, surface areas and mechanical properties suitable for different tissue engineering applications. Electrospun zein nanofibers mimic the extracellular matrix (ECM) structure, providing a high surface area for cell adhesion and proliferation. Zein microparticles can be used as injectable scaffolds or as building blocks for larger constructs. Zein films can serve as barrier membranes or as substrates for cell culture. The ability to tailor the scaffold architecture is crucial for guiding tissue regeneration. Zein scaffolds have been investigated for bone tissue engineering, cartilage regeneration, and skin wound healing. Studies have shown that cells can adhere, proliferate and differentiate on zein‐based scaffolds. Scientists demonstrated that human mesenchymal stem cells (hMSCs) adhered and proliferated well on electrospun zein scaffolds, suggesting their potential for regenerative medicine. The surface properties of zein, including its hydrophobicity and presence of specific amino acid sequences, can influence cell‐material interactions. Surface modification with bioactive molecules like cell adhesion peptides (e.g., RGD) can further enhance cell attachment and spreading. The porous structure of zein scaffolds allows for nutrient and waste exchange, supporting cell survival and growth. ZNPs can be incorporated into zein‐based scaffolds to provide controlled release of growth factors, promoting tissue regeneration. Scientists incorporated bone morphogenetic protein‐2 (BMP‐2) loaded zein microspheres into a porous zein scaffold for bone regeneration. The sustained release of BMP‐2 from the microspheres within the scaffold enhanced osteogenic differentiation of seeded cells. Encapsulating growth factors within ZNPs protects them from degradation and allows for their gradual release within the tissue engineering scaffold. This localised and sustained delivery can enhance cell recruitment, proliferation, differentiation and matrix deposition, leading to improved tissue repair and regeneration.

ZNPs can be utilised as carriers for imaging agents, enabling enhanced visualisation of biological processes. Researchers have explored the use of ZNPs to deliver contrast agents for various imaging modalities, such as magnetic resonance imaging (MRI) and fluorescence imaging. Scientists loaded superparamagnetic iron oxide nanoparticles (SPIONs) into zein nanoparticles for potential use as MRI contrast agents. The zein shell provided stability and biocompatibility to the SPIONs. Encapsulating contrast agents within ZNPs can improve their stability, prolong their circulation time, and enhance their accumulation at the target site, leading to improved image contrast and diagnostic accuracy. Surface modification with targeting ligands can further enhance the specificity of ZNP‐based contrast agents. ZNPs can serve as efficient delivery systems for active cosmetic ingredients, enhancing their penetration into the skin and providing controlled release. Their biocompatibility and biodegradability are also advantageous in this context. Zein itself exhibits some antimicrobial properties, and ZNPs can be used to deliver antimicrobial agents locally, potentially reducing systemic exposure and combating antibiotic resistance. Coatings based on zein nanoparticles incorporating antimicrobial agents have also been explored for medical devices.

Some studies have reported that zein micro‐ and nanoparticles can induce an immune response, including the production of anti‐zein antibodies. While generally low, this potential immunogenicity needs careful evaluation, especially for long‐term or repeated administration. Recent research suggests that ZNPs may not significantly influence innate immunity and inflammation, which is a positive indicator for their safety. Like other nanoparticles, ZNPs could potentially accumulate in certain organs, although studies suggest they are generally well‐tolerated in animals. Biodistribution studies are crucial to assess the extent of accumulation and potential long‐term effects. The primary toxicity concerns might arise from the encapsulated therapeutic agent or the materials used for surface modification rather than the zein itself. Thorough toxicological evaluation of the complete formulation is essential. In vitro studies have generally shown that ZNPs are non‐toxic to various cell lines. However, in vivo studies are crucial to confirm these findings in a complex biological system, considering factors like protein corona formation and interactions with the immune system. Toxicity might vary depending on the route of administration and the intended application (e.g., oral vs. intravenous delivery, use in tissue engineering scaffolds). The method of ZNP preparation, including the use of solvents or crosslinking agents, could introduce potential toxicity if residues are not adequately removed. Studies have generally indicated that zein nanoparticles exhibit low cytotoxicity in vitro. In vivo acute toxicity studies in animals have shown that ZNPs are generally safe at tested doses, with no significant mortality or abnormal behaviour observed. Subacute toxicity studies have also suggested that ZNPs are well‐tolerated over short periods, with no significant adverse effects on haematological or biochemical parameters. Research has focused on evaluating the immunotoxicity of ZNPs, with some studies suggesting a mild adaptive immune response but generally low impact on innate immunity.

## 3 Exploring the Methods of Manufacturing Zein Nanoparticles

Oleandro et al. [29] underscore the sustainable potential of ZNPs as nanocarriers for active chemicals, accentuating their biocompatibility and biodegradability. Garavand et al. [30] provide a thorough analysis of ZNPs in food packaging, examining their mechanical and barrier characteristics, and addressing safety issues related to nanotechnology. Liu et al. [31] examine the synthesis techniques and medicinal uses of ZNPs, highlighting their capacity to improve medication stability and bioavailability. Campos et al. [32] examine many synthesis strategies, including antisolvent precipitation and electrospraying, assessing their efficacy in generating stable nanoparticles for the encapsulation of bioactives exhibiting varied biological activities. These studies together highlight the many applications of ZNPs and their promise for future progress across several areas.

Several methods have been explored for the production of zein nanoparticles. (a) Antisolvent Precipitation: This is a common and relatively scalable method where zein is dissolved in a suitable solvent (e.g., ethanol) and then precipitated by the addition of a non‐solvent (e.g., water). Particle size can be controlled by adjusting parameters like flow rates, concentrations, and mixing conditions. (b) Emulsification‐Solvent Evaporation: This technique involves forming an emulsion of the zein solution in an immiscible solvent, followed by solvent evaporation to form nanoparticles. (c) Nanoprecipitation using Microfluidics: Microfluidic devices offer precise control over mixing and reaction conditions, leading to more uniform nanoparticles and potentially better scalability. (d) Spray Drying: This method involves atomising a zein solution into a hot gas stream, leading to rapid solvent evaporation and the formation of dry nanoparticles. It is a well‐established technique for large‐scale powder production.

## 4 Molecular Synthesis Techniques: A Deep Dive Into Ethanol‐Based Approaches and pH Modulation

The most conventional route for producing zein nanoparticles is to first dissolve the protein in ethanol and then trigger protein precipitation by finely adjusting the pH levels. This ethanol‐based approach is not only essential for controlling the particle size but also acts as a pivotal step for any further surface modifications, thereby allowing for customizable nanoparticles to suit specific end‐use applications. Furthermore, zein's inherent fluorescence properties serve as an invaluable asset for real‐time tracking and imaging procedures in scientific research [33].

## 5 Structural Intricacies: Helical Geometry and Its Molecular Interactions

Structurally, zein is characterised by a helical geometry stabilised by a complex network of hydrogen bonds. These hydrogen bonds connect nine homologous repeating units that are organised in an anti‐parallel manner. This particular configuration culminates in a slightly asymmetrical yet stable molecular structure, contributing to its unique biochemical properties [33]. It also exhibits globular features, akin to conventional proteins such as ribonuclease and insulin, which underscores its conformational adaptability [30].

## 6 Uniqueness in Amino Acid Profile: The Solubility Paradigm

One of the most compelling aspects of zein's structural architecture is its distinctive amino acid profile. The absence of amino acids like lysine, tryptophan, histidine, and arginine renders Zein distinctively different from most proteins, influencing its solubility in solvents and its interaction with other compounds [34].

## 7 Comprehensive Characterisation Metrics: Beyond Standard Measures

When it comes to characterising zein nanoparticles, several key parameters are evaluated, such as particle size, polydispersity index (PI), zeta potential and encapsulation efficiency. While particle size and zeta potential are typically quantified using photon correlation spectroscopy, electron microscopy offers nuanced insights into surface morphology. Sophisticated analytical techniques like Atomic Force Microscopy, Dynamic Light Scattering, fluorescence spectroscopy, UV spectroscopy, Circular Dichroism and differential calorimetry have been employed to explore the structural transitions and stability metrics in zein nanoparticles [34].

## 8 Temperature‐Sensitive Morphological Changes: Thermal Treatment Analysis

The thermal behaviour of zein nanoparticles has been a subject of extensive research. Studies have shown that heating Zein dissolved in 70% ethanol at varying temperatures (75°C, 85°C and 95°C) and for different time intervals (15, 30 and 45 min) led to a series of transformative changes. These include alterations in particle size distribution, molecular geometry (α‐helix and β‐sheet ratios) and thermal stability. Notably, thermal treatment at lower temperatures yielded particles with a more uniform size, whereas higher temperatures led to aggregation and structural distortions [34].

## 9 pH Sensitivity and Its Multi‐Faceted Impact

Zein's sensitivity to pH alterations is accentuated by its isoelectric point of 6.2. Experiments have shown that changes in pH can induce significant conformational shifts, affecting the protein's structural, rheological and antioxidant properties. SDS‐PAGE analyses conducted at varying pH levels (acidic, neutral and alkaline) indicated that the molecular weight and polymerisation state remained largely unchanged, yet the antioxidant capacity was considerably influenced [35].

## 10 Recent Innovations: Curcumin Encapsulation Through Alkaline Deamination

One of the most recent advancements in zein nanoparticle research involves its encapsulation capabilities when treated with curcumin under alkaline conditions. This alkaline deamination treatment was shown to enhance the solubility, stability and antioxidant properties of zein, thereby widening its applicability in various sectors [35].

With continued advancements in research methodologies and a renewed focus on sustainability, zein nanoparticles hold immense promise for commercial ventures, particularly as researchers find ways to minimise environmental impact by reducing or recycling solvents. The intricate balance of structural stability, encapsulation capabilities and functional versatility makes zein nanoparticles a fascinating subject for future exploration and commercial exploitation.

## 11 Advancements in Zein‐Based Nanoparticles: Pioneering Strategies for Drug Delivery Systems and Functionalised Emulsions

**FIGURE 1:**
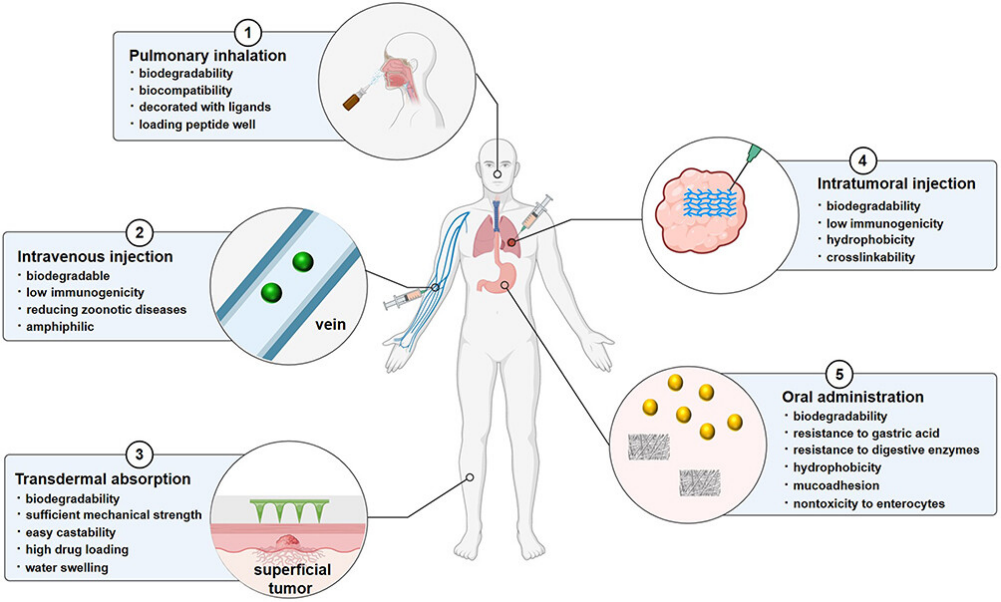
Routes of administration for zein carriers and key properties of zein relevant to each administration route [Adapted from reference: [36]].

The burgeoning field of nanomedicine is experiencing transformative advancements with the incorporation of Zein‐derived nanoparticles, an environmentally‐friendly and biodegradable class of nanocarriers originating from plant proteins. Zein offers a cost‐effective alternative to other materials due to its natural abundance (Figure 1).

### 11.1 Precision Engineering of Zein Nanoparticles: The Interplay of Alcohol Concentration and pH

The inherently hydrophobic and water‐repellent properties of zein call for specialised preparation methods. The standard practice of utilising a pH‐sensitive nanoprecipitation technique offers a balanced method for particle synthesis, calling for an alcohol concentration in the range of 55%–100%. Notably, the nanoparticle dimensions are inversely related to the alcohol concentration, providing a tool for targeted size manipulation [30].

The role of pH is particularly crucial in zein nanoparticle synthesis. The electrical charge of the particles is contingent on the pH level due to zein's isoelectric point at 6.8—negative charges occur when pH exceeds the isoelectric point, and positive charges result when it falls below. This charge polarity has direct ramifications on the final nanoparticle dimensions [37]. These complex dynamics warrant comprehensive research to fully understand the governing principles.

### 11.2 Stabilisation Agents: Limitations and Innovations

Lecithin and Pluronic F68 serve as the cornerstone stabilising agents. While lecithin demonstrates an affinity for zein, its solubility in alcohol compromises its efficacy. In contrast, Pluronic F68 consistently fosters the production of stable zein nanoparticles that are free from aggregation [38].

### 11.3 Next‐Generation Coatings and Versatile Encapsulation Capabilities

Polyethylene Glycol (PEG)‐coated zein nanoparticles stand as breakthrough innovations, particularly in the realm of oral drug administration. Following an initial desolvation phase, a subsequent PEG coating is applied. Electron microscopy and FTIR analysis subsequently confirm the successful coating process [35]. Uniquely, these PEG‐coated particles have the potential to accommodate a wide array of substances, from hydrophobic molecules to hydrophilic macromolecules [39]. These nanoparticles extend the scope of bioavailability, and impressively, they achieve this feat without necessitating the incorporation of novel chemical constituents [40].

### 11.4 Cutting‐Edge Fabrication Technologies: A Focus on SEDS

The Solution Enhanced Dispersion by Supercritical CO_2_ (SEDS) technique represents a novel frontier in optimising zein nanoparticles. Key variables such as nozzle design and CO_2_ flow rates influence the morphology, distribution and velocity fields of the nanoparticles. Computational Fluid Dynamics (CFD) provides the analytical framework for understanding these complex dynamics [41].

### 11.5 Unpacking Zein Extraction Techniques and Structural Insights

Typically, zein is extracted from distillers dried grains with solubles (DDGS) employing diverse techniques like the single column extraction methodology. Following the drying stage, zein yields approximately 30.7% [27]. X‐ray diffraction methods further contribute crucial insights into the amorphous nature of the resulting zein nanoparticles [42].

### 11.6 The Potent Impact of Zein on Antioxidant‐Stabilised Emulsions

The interaction between gallic acid (GA) and zein nanoparticles in Pickering emulsions exhibits a dynamic equilibrium. A direct correlation emerges between increased zein nanoparticle concentration and elevated levels of GA, thereby enhancing the oxidative stability of the emulsions. Zein not only serves as an interfacial stabiliser but also significantly amplifies antioxidative capabilities [43].

**FIGURE 2:**
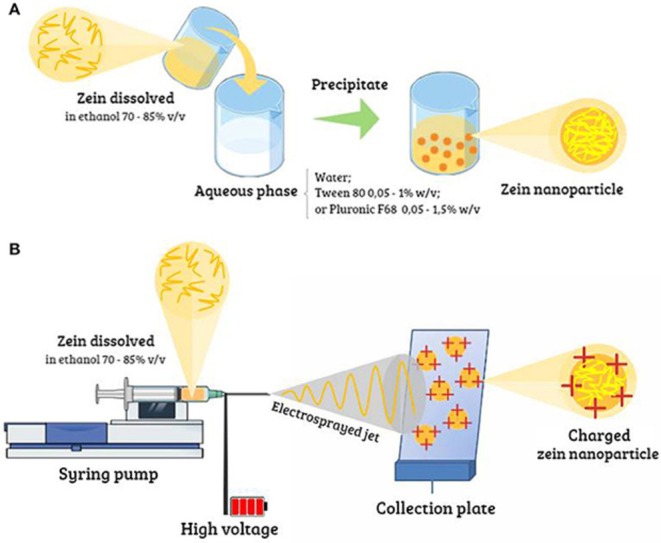
Preparation of zein nanoparticles: (A) antisolvent precipitation/liquid–liquid dispersion/phase separation techniques and (B) electrohydrodynamic atomisation [Adapted from reference: [44]].

**FIGURE 3:**
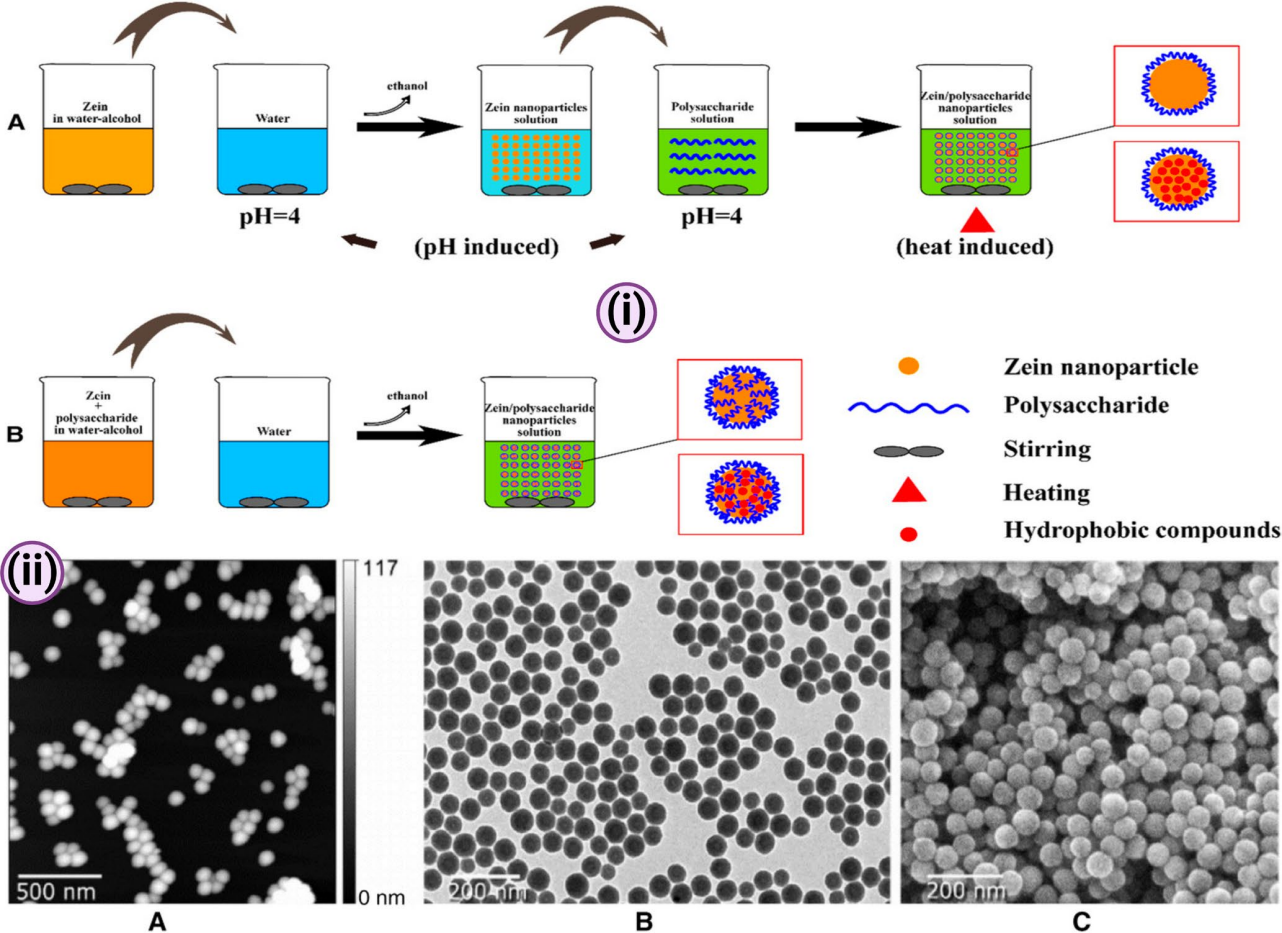
(i) The methods of preparation of zein/polysaccharide nanoparticles: (A) pH‐ and heat‐induced antisolvent precipitation; (B) antisolvent coprecipitation [Adapted from reference [45]]; (ii) The morphological observation of nanoparticles by (A) AFM, (B) TEM and (C) SEM. [Adapted from reference [46]].

The transformative potential of zein nanoparticles is beckoning researchers and clinicians alike, offering innovative approaches from enhanced drug delivery mechanisms to functionalised emulsions. With continued research to elucidate the intricacies in preparation techniques and potential applications, zein nanoparticles promise a host of groundbreaking solutions in nanomedicine (Figures 2 and 3).

## 12 Development and In‐Depth Analysis of Zein Based Nanoparticles

Zein/Polysaccharide nanoparticulate systems have emerged at the forefront of advanced food and pharmaceutical research, bringing together the strengths of zein proteins and various polysaccharides (Figure 3). Their potential spans across several key functionalities, including:

- Extending the applicability of hydrophobic substances across diverse food and pharmaceutical mediums
- Enhancing the protection of bioactive ingredients against degrading environmental factors
- Improving the bioavailability of encapsulated ingredients through a controlled gastrointestinal tract (GIT) release mechanism
- Fine‐tuning encapsulation efficiencies for maximum benefit

## 13 Alginate and Propylene Glycol Alginate (PGA): Biopolymer Virtuosos in Food Technology

**FIGURE 4:**
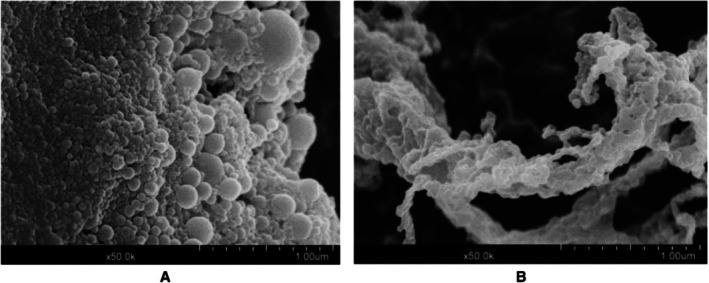
SEM images of (A) zein nanoparticles and (B) zein/PGA nanoparticles. [Reprinted from [47]].

Derived from brown seaweed, alginate has firmly established its position within the food industry. As an anionic linear polysaccharide, its prowess as a thickening agent, stabiliser, and emulsifier has made it indispensable. The fascinating gelation properties it exhibits in the presence of divalent ions, especially Ca^2+^, have further expanded its applications in culinary and pharmaceutical innovations. In tandem, Propylene Glycol Alginate (PGA) stands out, offering exclusive solubility attributes in select aqueous environments, widening the scope for creating specialised gels (Figure 4). Zein/Alginate nanoparticle (ZAN) development largely leans on the pH‐driven antisolvent precipitation method. Such advanced techniques bolster the nanoparticles' resilience against a plethora of challenges, from fluctuating pH levels to thermal variabilities and even unpredictable ionic strengths. Scholarly literature increasingly recognises ZANs as key vehicles in the realm of nano delivery.

## 14 Plant‐Derived Pectin

Pectin, sourced meticulously from plant cell walls, is a paragon in the world of anionic structural polysaccharides. The gelling potential and thickening properties it possesses can be attributed to the delicate esterification processes of galacturonic acid units. When integrated into the zein/pectin nanoparticle (ZPN) framework, the resultant structure boasts heightened stability. However, the susceptibility of ZPNs to intense ionic environments warrants further research. In scientific circles, ZPNs are frequently cited for their superior encapsulation capabilities, offering enhanced bioactivity and optimal dispersibility for encapsulated ingredients.

## 15 Chitosan Chronicles: From Marine Exoskeletons to Precision Delivery

Chitosan, a natural biopolymer derived from chitin found in crustacean shells, has emerged as a promising material for drug delivery systems [48, 49]. Its biocompatibility, biodegradability, and low toxicity make it an ideal candidate for various pharmaceutical applications [50]. Chitosan nanoparticles have shown particular promise in oral drug delivery, overcoming challenges such as low solubility and poor absorption [51]. These nanoparticles can be synthesised through various methods, including ionic cross‐linking and precipitation, and can be functionalised for targeted delivery [49]. Chitosan‐based nanomaterials have demonstrated effectiveness in multiple drug delivery applications, including ocular, pulmonary, nasal and cancer therapies [48, 50]. Recent advancements have focused on developing chitosan‐based nanocomposites for gene delivery, wound healing and antimicrobial treatments [49]. The versatility and efficacy of chitosan nanoparticles highlight their potential to revolutionise drug delivery systems.

Chitosan's journey, from the humble origins of chitin often found in crustacean exoskeletons, to a coveted biopolymer, has been nothing short of transformative. With its plethora of biological benefits, it is a prime contender in targeted drug delivery platforms, especially favouring oral delivery mechanisms. Zein/Chitosan nanoparticles (ZCNs) [52] are exemplary of the meticulous engineering that underpins nanoparticle design—safeguarding encapsulated compounds and ensuring their systematic release in the GIT [52]. Recent explorations have led to the inception of zein/oligochitosan nanoparticles (ZOCNs), with these next‐gen nanoparticles boasting improved water solubility and an amplified defence strategy against external environmental threats.

## 16 Polysaccharide Mediators in Nanoparticle Synthesis

Polysaccharide mediators play a crucial role in the synthesis of nanoparticles, particularly in the context of ‘green synthesis’ methods. These natural biopolymers offer a sustainable and biocompatible alternative to traditional chemical and physical synthesis routes, which often involve toxic reagents and harsh conditions. Polysaccharides primarily act as both reducing agents and stabilising agents in nanoparticle synthesis. Polysaccharides contain abundant hydroxyl groups, hemiacetal reducing ends, and other functional groups that can reduce metal ions to their zero‐valent metallic nanoparticle form. This reduction process often involves the oxidation of the polysaccharide. The specific mechanism can vary depending on the polysaccharide and reaction conditions, sometimes involving glycosidic bond breakage or the formation of highly reactive enediols under alkaline conditions. After reduction and nucleation, polysaccharides adsorb onto the surface of the nascent nanoparticles, preventing agglomeration and controlling their size, shape and stability. This ‘capping’ effect is due to the steric hindrance and electrostatic repulsion provided by the polysaccharide chains. The presence of numerous hydroxyl, carboxyl and amine groups on the polysaccharide backbone facilitates strong interactions with the nanoparticle surface. Polysaccharide capping improves the colloidal stability of nanoparticles, preventing aggregation and maintaining their dispersed state. Polysaccharides offer a wide range of structures and functional groups, allowing for tailored synthesis of nanoparticles with diverse properties and applications. They can also be easily modified chemically or enzymatically to enhance specific functionalities. Polysaccharides are natural polymers, generally non‐toxic, and readily biodegradable, which is highly advantageous for biomedical applications. Many polysaccharides possess intrinsic biological activities (e.g., anti‐inflammatory, antioxidant, antimicrobial, immunomodulatory) that can confer additional therapeutic benefits to the resulting nanoparticles. Some polysaccharides have specific binding affinities for certain receptors on cells, enabling targeted drug delivery (Table 3).

**TABLE 3:** Polysaccharide mediators in nanoparticle synthesis.

Polyose	Derivation	Key features and interactions	Applications
ι‐Carrageenan	Sulphated polysaccharide	High charge density due to sulphate groups. Interactions: Electrostatic, hydrogen bonding, hydrophobic	Widely used in the food industry. Formation of core‐shell zein/carrageenan nanoparticles.
HPMC	Derived from cellulose	Water‐soluble polymer with swelling/dissolution properties.	Modulate drug release. Formulation of zein/HPMC nanoparticles.
Tea polysaccharides (TPs)	Extracted from green tea	Protein‐bounded acidic polysaccharides.	Creation of nanoparticles sustaining release of compounds and maintaining them in an amorphous state.
Gum Arabic (GA)	Polypeptide glycated on the saccharide chain	Broad pH solubility. Excellent emulsifying properties.	Formation of zein/GA nanoparticles. Potential applications in encapsulation.
Hyaluronic acid (HA)	Comprised of D‐glucuronic acid and N‐acetyl‐glucosamine	Biocompatible. Interactions: Electrostatic attraction, hydrogen bonding, hydrophobic effects.	Zein/HA nanoparticles fabrication using antisolvent coprecipitation.
Rhamnolipids	Surface‐active glycolipids	Contains hydrophilic rhamnose units and hydrophobic fatty acid chain.	Zein/Rhamnolipids nanoparticles encapsulating curcumin.
Chondroitin sulphate (CS)	Linear polysaccharide with repeating disaccharide units	Inherent negative charge. Interactions: Electrostatic, hydrogen bonding, hydrophobic.	Zein/CS nanoparticles exhibit great stability to pH and heat treatment.
Soybean polysaccharide (SP)	By‐product from tofu and soybean protein processing	Highly water‐soluble, low‐viscosity, heat‐stable, negatively charged.	Zein/SP nanoparticles known for encapsulation efficiency and enhancing photochemical stability.
Agar	Structural carbohydrate in cell walls of specific algae	Exceptional gelation properties. High solubility in hot water.	Formation of coacervates or nanoparticles made of zein and agar.

### 16.1 ι‐Carrageenan

Within the realm of sulphated polysaccharides, ι‐Carrageenan stands distinguished. Its unique structural composition, replete with sulphate groups, grants it a pronounced charge density, a feature that has not escaped the discerning eye of food scientists. Extensive analyses have revealed its potential in the food industry, especially considering its intricate interactions with proteins and the subsequent effects on gelation processes. Particularly, core‐shell zein/carrageenan nanoparticles, formulated via the antisolvent precipitation technique, exhibit noteworthy stability attributes. The role of calcium ions in cross‐linking, coupled with multi‐faceted interactions—electrostatic, hydrogen bonding and hydrophobic—facilitates the structural integrity of these nanoparticles.

### 16.2 Hydroxypropyl Methylcellulose (HPMC)

HPMC's lineage traces back to cellulose, an omnipresent organic compound. Its amenability to swell and dissolve in aqueous environments earmarks it as an exemplary candidate for modulating drug release kinetics. Pioneering research has demonstrated that Zein/HPMC nanoparticle constructs, known as ZHNs, have the prowess to augment the dissolution profiles of traditionally water‐insoluble therapeutic agents. Surfactants, when introduced, accentuate this property, thereby minimising crystallisation of the drug entities.

### 16.3 Tea Polysaccharides (TPs)

With their origins rooted in green tea, TPs exude a unique biochemical signature, predominantly protein‐bound acidic polysaccharides. Their synergistic collaboration with zein leads to the genesis of nanoparticles that not only modulate the release dynamics of encapsulated entities but also preserve their amorphous structural integrity.

### 16.4 Gum Arabic (GA)

The versatility of GA is encapsulated in its commendable solubility across a broad pH spectrum and its inherent emulsifying acumen. This prowess is largely attributed to the glycated polypeptide on its saccharide backbone. Zein/GA nanoparticles, however, demand caution when exposed to elevated thermal conditions due to potential structural compromises.

### 16.5 Hyaluronic Acid (HA)

HA, a polysaccharide consisting of intricately arranged D‐glucuronic acid and N‐acetyl‐glucosamine units, finds resonance in the medical community for its biocompatibility and host of physiological advantages. In the realm of nanoparticle fabrication, zein/HA nanoparticles are synthesised leveraging the antisolvent coprecipitation methodology, emphasising the role of electrostatic attractions, hydrogen bonding and hydrophobic interactions in nanoparticle stabilisation.

### 16.6 Rhamnolipids

Recognised as potent glycolipids, rhamnolipids are pivotal in reinforcing the stability of an array of nanoparticles. Their chemistry enables the formulation of zein/rhamnolipids nanoparticles, adept at encapsulating compounds such as curcumin. The solubility attributes of these entities, especially in strong alkaline environments, play a cardinal role in their formulation.

### 16.7 Chondroitin Sulphate (CS)

CS, architecturally a linear polysaccharide, possesses inherent negative charges courtesy of its constituent disaccharide units. Zein/CS nanoparticles are sculpted with spherical precision, deriving their stability from a harmonious interplay of electrostatic, hydrogen bonding and hydrophobic interactions.

### 16.8 Soybean Polysaccharide (SP)

A by‐product of tofu and soybean protein processing, SP is acknowledged for its prodigious solubility and thermal resilience. Zein/SP nanoparticles, under the aegis of optimal pH conditions, have manifested capabilities that extend beyond encapsulation efficiency to bolstering the photochemical stability of the compounds they encase.

### 16.9 Agar

Hailing from the cell walls of specific algae, agar's propensity for gelation is unparalleled. In the context of nanoparticle synthesis, zein and agar converge to form coacervates, the microstructural dynamics of which are significantly influenced by the ionic strength of the solutions they inhabit.

## 17 Unique Attributes of Zein Nanoparticles‐Innovations in Zein Nanoparticle‐Mediated Gene Therapy for Cancer Treatment

Zein nanoparticles, composed of a prolamin protein originating from corn, have marked themselves as paramount contenders in the arena of nanocarriers. Specifically designed for low toxicity, high binding affinities for a variety of drug compounds and sourced from abundant renewable resources, zein nanoparticles are on the vanguard of medical applications, including but not limited to oncology.

### 17.1 Biochemical Advantages: Implications for Drug Pharmacokinetics and Bio‐Distribution

Nanocarriers such as zein nanoparticles amplify the efficacy of drug delivery by virtue of their elevated surface area‐to‐volume ratios. These biochemical advantages play a pivotal role in optimising the biodistribution and pharmacokinetics of therapeutic agents complexed with zein. The enhanced permeability of such complexes positions zein nanoparticles as robust vehicles for gene delivery systems. Importantly, these nanoparticles have earned the GRAS (Generally Recognised as Safe) designation, consolidating their potential for broad medical applications [48].

### 17.2 Hepatocellular Carcinoma (HCC): A Novel Approach Using Zein‐Enabled Gene Therapy

In the field of liver oncology, particularly Hepatocellular Carcinoma (HCC), gene therapy has been envisioned as a transformative therapeutic avenue. The efficiency of gene therapy is fundamentally tethered to the vector's design, along with the precision and efficacy in gene delivery to targeted cellular sites [49]. Zein nanoparticles, when loaded with specific anti‐cancer agents such as PTEN (Phosphatase and Tensin Homologue) and TRAIL (Tumour Necrosis Factor‐Related Apoptosis‐Inducing Ligand), have been postulated to offer improved therapeutic outcomes.

### 17.3 Molecular Interplay: The PTEN and TRAIL Synergy

PTEN is renowned for its tumour‐suppressive capabilities, particularly in its capacity to thwart proliferation, migration and invasive attributes of HepG2 liver cell lines. It performs these functions through its intrinsic phosphatase activity, which antagonistically regulates cell growth and survival mechanisms [50]. TRAIL's role in apoptosis becomes significantly more effective when co‐delivered with PTEN via zein nanoparticles. This results in upregulation of markers such as p53, VEGF and MMP‐2 at the mRNA level, thereby reviving apoptotic pathways and arresting cancer progression [50].

### 17.4 Luteolin‐Zein Complex: Emerging Solutions in Colorectal Oncology

Luteolin, a flavonoid renowned for its anti‐cancer and anti‐inflammatory potency, has seen its therapeutic efficacy hampered due to its limited solubility in aqueous solutions. Zein nanoparticles, especially when used in conjunction with sodium caseinate, have proven to significantly enhance the bioavailability and thus, therapeutic utility of luteolin. Particle size in these formulations is crucial; ranging between 70 and 280 nm, these nanoparticles enable highly specific targeting of essential biological sites [50].

### 17.5 Breast Cancer: Pioneering Treatment Modalities With Zein

Exemestane and Resveratrol, two drugs frequently used in breast cancer therapy, suffer from pharmacological limitations owing to their poor solubility and permeability. Zein‐encapsulation has proven to be a game‐changer, augmenting the cytotoxic capabilities of these drugs against breast cancer cells [51].

### 17.6 Drug Delivery Kinetics: Adherence to the Hixson‐Crowell Model

When it comes to oral administration, the luteolin‐encapsulated zein nanoparticles align closely with the well‐established Hixson‐Crowell model, offering new avenues for optimising drug delivery systems. The zein nanoparticle platform stands as an extraordinary candidate for disrupting traditional paradigms in cancer therapy. It offers a multiplicity of advantages, from optimising drug delivery and enhancing bioavailability to enabling more precise gene therapies. As research in this domain continues to evolve, zein nanoparticles have the potential to become integral elements in the next generation of cancer treatment protocols.

### 17.7 Cancer Treatment Using Zein Nanoparticles

**FIGURE 5:**
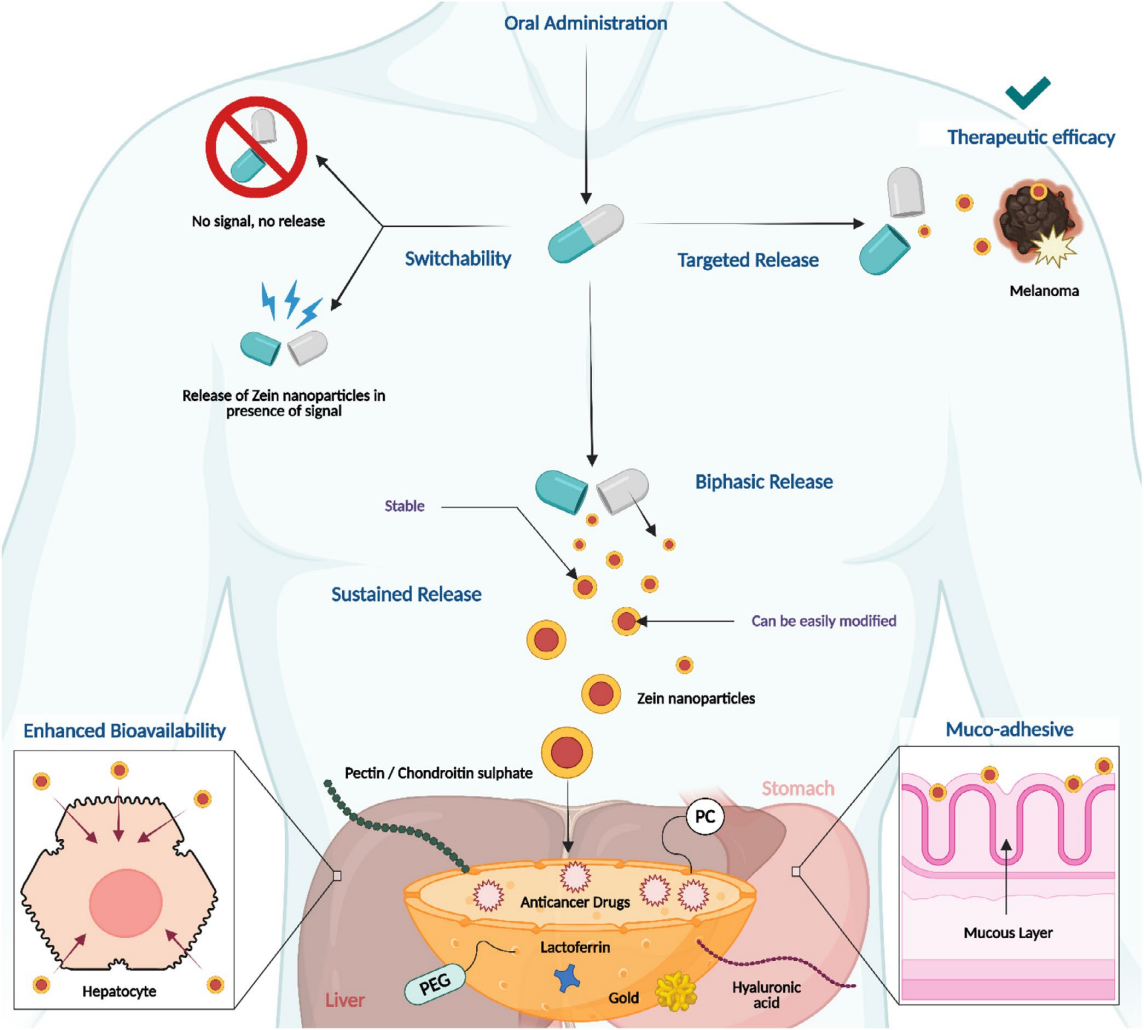
Properties of zein nanoparticles making them suitable for targeted delivery of chemotherapeutic agents against a wide range of cancers.

Even though the clinical translation of zein‐based nanoparticles for cancer therapy is still nascent, observations made from experiments performed on cell lines and animal models suggest that zein nanoparticles have extraordinary drug delivery capabilities and therapeutic efficiency owing to their stability, adhesion to mucous, sustained and targeted release, modifiability and enhanced bioavailability (Figure 5).

Identifying personalised therapeutics and patient‐specific ligands for zein nanoparticles used in cancer therapy involves a multilayered approach based on molecular profiling of a patient's tumour, utilising advanced OMICS technologies. This begins with genomic sequencing (exome or transcriptome sequencing, including single‐cell analysis) to identify unique mutations, polymorphisms, gene amplifications or aberrant expression specific to the patient's cancer cells, even revealing intra‐tumour heterogeneity. Complementary proteomics then identify overexpressed receptors or mutated proteins. Based on these insights, personalised therapeutics are identified; for instance, existing targeted therapies for specific oncogenic mutations, or designs for personalised mRNA vaccines against unique tumour neoantigens, or siRNA for gene silencing. Simultaneously, patient‐specific ligands are discovered by analysing surface receptor profiles from the OMICS data and employing techniques like phage display or aptamer selection or computational methods to find high‐affinity binders. Once both the personalised therapeutic cargo and the specific ligand are validated, the zein nanoparticle is modified and functionalised. The therapeutic agent is encapsulated within the zein core, and the patient‐specific ligand is covalently conjugated to the nanoparticle's surface using established bioconjugation chemistries. This integrated approach allows for the synthesis of highly tailored and personalised nanomedicines, precisely targeting and delivering therapies based on an individual patient's signatures and biomarkers related to cancer. Although more comprehensive investigations are needed, zein nanoparticles can advance personalised cancer therapy by precisely targeting the unique characteristics of an individual's tumour, moving beyond general approaches. This can involve active targeting with patient‐specific ligands, where zein nanoparticles are customised with biomarkers like specific antibodies or peptides identified directly from a patient's cancer cells, ensuring highly precise binding and decreased side effects. Moreover, zein nanoparticles can be engineered and functionalised for tumour microenvironment–responsive rapid drug release, delivering their therapeutic payload specifically when triggered by individual tumour conditions such as acidic pH, unique enzyme overexpression or changed redox potential. Zein's versatility, such as switch‐ability, muco‐adhesion, and sustained biphasic release of cargo, also allows for the delivery of personalised therapeutics, including patient‐specific mRNA vaccines against unique tumour antigens or siRNA and CRISPR components to silence genes crucial for an individual's tumour survival or chemoresistance, making it a good choice for precision cancer therapy.

### 17.8 Delivery of pH‐Responsive Anticancer Drugs

Scientists have explored an innovative path of utilising zein nanoparticles as a base material for encapsulating cancer drugs and enhancing their pH‐responsive delivery capabilities [53, 54, 55, 56]. Scientists have proposed the idea of combining zein nanoparticles with tannic acid and a range of different metal ions such as copper, zinc and iron. The inclusion of these ions was critical as they influence the stability and dissolution rate of the zein nanoparticles. The central premise of their research was rooted in the understanding that different tissues in the body have varying pH levels, and more importantly, that cancerous tissues often present a distinctively acidic microenvironment compared to their healthy counterparts [53]. This differential in pH levels can be leveraged to trigger the selective release of anticancer drugs, thus improving targeting and minimising collateral damage to healthy cells. In their experimental setup, the team synthesised nanoparticles through a solvent evaporation method, where zein proteins were dissolved in ethanol, combined with tannic acid, and then various metal ions were added. Following this, a comprehensive set of in vitro tests was conducted to assess the nanoparticles' stability, dissolution rates, and release profiles under varying pH conditions [53].

One of the most pivotal findings of their research was that the rate of disintegration of the zein nanoparticles could be finely tuned by altering the types and concentrations of the metal ions used in the formulation. By manipulating these variables, researchers successfully engineered nanoparticles that disintegrated more quickly in acidic environments, thus making them more suitable for targeted drug release in cancerous tissues. Another crucial discovery was that the tannic acid acted as a stabilising agent, forming a chelate with the metal ions. This interaction further optimised the nanoparticles for their pH‐responsive behaviour. Tannic acid not only increased the overall stability of the nanoparticles but also offered additional control over the drug release rates. The in vivo studies substantiated these in vitro findings. They observed that the engineered zein nanoparticles showed a substantially higher accumulation in tumour tissues compared to conventional formulations. This ultimately manifested in enhanced therapeutic outcomes, thereby validating the efficacy of their pH‐responsive drug delivery system [53]. Overall, such works shine as a beacon for those interested in leveraging the unique properties of zein nanoparticles for targeted, pH‐responsive drug delivery in cancer therapy. Their meticulous experimental design, comprehensive testing and insightful conclusions make their research a seminal contribution to this burgeoning field [53, 54, 55, 56].

### 17.9 Doxorubicin‐Loaded Hydrogels

To tackle the well‐documented issue of cytotoxic side‐effects associated with doxorubicin, a chemotherapy medication widely used for various types of cancer, Priyanka and her colleagues developed an innovative approach involving zein nanoparticles and pectin hydrogels [57]. Doxorubicin is an effective but also notably toxic drug; it does not discriminate well between cancerous and healthy cells, leading to damaging effects on normal tissues. This has long been a point of concern in cancer therapy. Priyanka's team sought to address this problem by encapsulating doxorubicin within zein nanoparticles, which were then incorporated into pectin hydrogels. The rationale was that this complex would enable more controlled, targeted delivery of the drug, thereby reducing toxicity. The team synthesised zein nanoparticles loaded with doxorubicin through a solvent evaporation technique, similar to what many other scientists have employed. These nanoparticles were then mixed into a pectin hydrogel matrix, resulting in a stable composite material capable of encapsulating and subsequently releasing doxorubicin. The pectin hydrogels were selected for their biocompatibility and hydrophilic nature, which further assisted in controlled drug release [57]. In their experiments, Priyanka's team used HeLa cells, a human cell line derived from cervical cancer cells, as the target. A series of in vitro assays were performed to examine the efficiency of drug delivery and the potential cytotoxic effects on both cancerous and healthy cells. They also tested the hydrogels under different environmental conditions to understand how temperature, pH and other factors might impact drug release rates [57].

A significant outcome of their work was the demonstration that the zein‐pectin hydrogel composites allowed for more gradual and controlled release of doxorubicin compared to conventional delivery methods. This had a dual advantage: it maintained the drug's efficacy in attacking cancer cells while significantly reducing its impact on non‐targeted healthy cells. Furthermore, the hydrogels themselves provided an added layer of control. The interaction between the pectin hydrogels and zein nanoparticles led to a form of ‘gated’ release of the drug. Essentially, the hydrogel acted as a barrier that could be manipulated to either hold the drug or allow its passage, offering an additional layer of precision in drug delivery [57].

The team also carried out in vivo studies to validate the in vitro results, confirming that the hydrogel composites were indeed effective in delivering doxorubicin specifically to cancerous cells while leaving healthy cells largely unharmed. This was a crucial step in validating the potential of their system for clinical applications [57]. In summary, Priyanka and her team's research provides a promising pathway for enhancing the therapeutic index of doxorubicin. By using a complex of zein nanoparticles and pectin hydrogels, they achieved a more controlled and targeted drug delivery system, which shows significant potential to reduce the collateral damage typically associated with this powerful anticancer drug [57].

### 17.10 Treatment of Prostate Cancer

**FIGURE 6:**
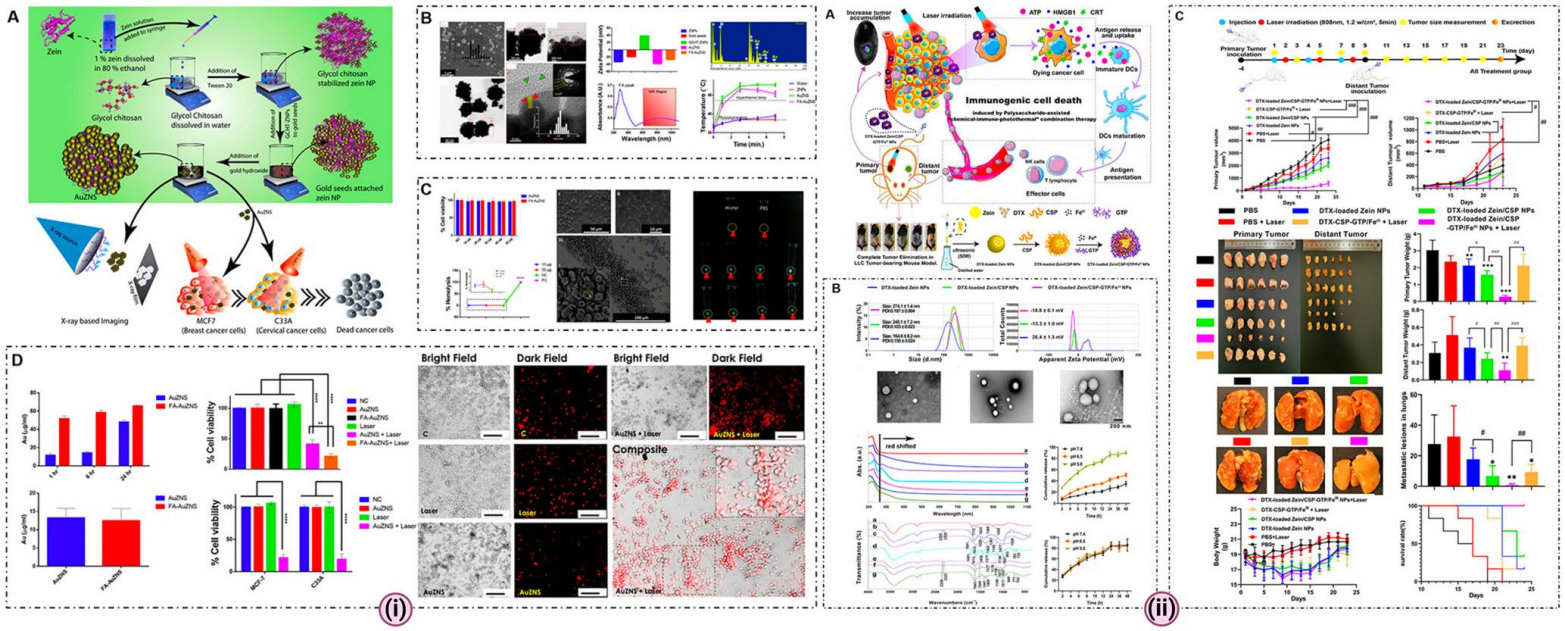
(i) (A) An illustration depicting the environmentally friendly synthesis process and subsequent utilisation of AuZNS (gold‐coated zein nanoshells) for imaging‐guided plasmonic photothermal therapy. The associated micrographic images present the size distribution of the synthesised AuZNS, and Zeta potential measurements are included to characterise their surface charge; (B) Elemental analysis results demonstrate the elemental composition of AuZNS, and the absorbance spectrum highlights their optical properties. Moreover, the photothermal transduction capability of AuZNS is illustrated; (C) biocompatibility and hemolysis studies are showcased to assess the safety of AuZNS. X‐ray images of negative controls, AuZNS samples and Omnipaque (a contrast agent) are displayed at varying concentrations, offering a comparison of their imaging properties; (D) Qualitative analysis is presented, focusing on non‐targeted photothermal therapy conducted on C33A cells using propidium iodide staining. The uptake study examines the cellular internalisation of AuZNS, distinguishing between targeted and non‐targeted photothermal therapy approaches. Statistical significance is denoted by asterisks, with ‘**p < 0.01’ and ‘****p < 0.0001’ indicating the levels of statistical significance. [This figure and associated data are adapted with permission from a study titled ‘Facile synthesis of plasmonic zein nanoshells for imaging‐guided photothermal cancer therapy’ by Chauhan DS et al., published in the journal ‘Materials Science and Engineering C: Materials for Biological Applications’ in 2018 and copyrighted by Elsevier [59]]. (ii) (A) Schematic diagram of the ‘all‐in‐one’ and ‘one‐for‐all’ nanoplatform for combined ‘chemo−immuno−photothermal’ therapy. (B) Fabrication and characterisation of DTX‐loaded zein/CSP‐GTP/Fe (III) NPs and the in vitro drug release. (C) In vivo therapeutic efficacy of various treatments on 4T1 tumour‐bearing mice. *p < 0.05; **p < 0.01; ***p < 0.001 compared to control. #, *p* < 0.05; ##, *p* < 0.01; ###, *p* < 0.001. [Reprinted with permission from [60]].

Han Lee and his team were particularly interested in improving the delivery of docetaxel, a chemotherapy medication commonly used for treating prostate cancer. Docetaxel is known for its efficacy but, like many chemotherapeutic agents, its application is often limited by poor selectivity and systemic toxicity [58]. To mitigate these challenges, the team developed zein nanoparticles that were chemically conjugated with chondroitin sulphate, a biological polymer. Chondroitin sulphate was specifically chosen due to its affinity for certain receptors commonly found on prostate cancer cells. By conjugating the zein nanoparticles with chondroitin sulphate, they aimed to increase the targeted delivery of docetaxel to cancer cells while minimising exposure to healthy cells [58] (Figure 6). The nanoparticle synthesis involved multiple stages, including the encapsulation of docetaxel within the zein nanoparticles and subsequent conjugation with chondroitin sulphate. The researchers employed various techniques such as dynamic light scattering and electron microscopy to assess the size, morphology, and colloidal stability of the nanoparticles [58]. A primary focus of their work was on understanding the pharmacokinetic behaviour of these nanoparticles, as well as their cellular uptake mechanisms. Extensive in vitro tests were conducted to validate that the zein‐chondroitin sulphate nanoparticles maintained their stability in various physiological conditions and were successfully taken up by prostate cancer cells. They also evaluated potential cytotoxicity and off‐target effects through a series of assays [58].

Their results indicated a strong colloidal stability of the nanoparticles, a critical property to ensure they remain intact in the physiological conditions they would encounter in the body. Importantly, their work also revealed enhanced cellular uptake, showing that the chondroitin sulphate conjugation did indeed facilitate more efficient targeting of prostate cancer cells [58]. In addition to in vitro testing, the team also carried out in vivo studies using animal models to validate the pharmacokinetic behaviour of the nanoparticles. They found that the zein‐chondroitin sulphate nanoparticles showed excellent circulation times, allowing for sustained release of docetaxel. Furthermore, the conjugated nanoparticles demonstrated improved drug delivery to prostate cancer cells, reducing the dosage required for effective treatment, and thereby also minimising systemic toxicity [58]. A major highlight of Han Lee's research was that it not only proved the concept of targeted drug delivery for treating prostate cancer but also provided a roadmap for optimising the pharmacokinetic properties of zein‐based nanoparticles. By carefully selecting chondroitin sulphate as the conjugate, they enhanced the precision of drug delivery, thereby offering a pathway for reducing the side effects commonly associated with chemotherapy [58].

In inference, this groundbreaking research laid down an important framework for the use of zein nanoparticles in cancer therapy, specifically in the context of prostate cancer. Their work suggests that chondroitin sulphate‐conjugated zein nanoparticles have the potential to be a game‐changer in making docetaxel‐based chemotherapy more targeted and less toxic [58].

Dong and his team worked on a comprehensive study where they integrated doxorubicin with zein nanoparticles and assessed drug release profiles under different pH conditions [61]. Their aim was to develop a nanoparticle‐based system that could release the drug over an extended period. The study concluded that their formulation was successful in providing controlled, long‐term release of doxorubicin, thereby increasing the therapeutic window.

### 17.11 Treatment of Metastatic Prostate Cancer

Thapa and colleagues developed zein nanoparticles with the goal of co‐delivering two potent drugs, vorinostat and bortezomib, targeting metastatic prostate cancer [62]. Vorinostat is an HDAC inhibitor that affects gene expression, and bortezomib is a proteasome inhibitor; both have demonstrated anti‐cancer properties. Their focus on dual‐action drugs was groundbreaking, given that metastatic prostate cancer poses a significant challenge due to its aggressive nature and tendency to spread to other parts of the body. The research involved intricate nanoparticle design and characterisation, followed by exhaustive in vitro and in vivo tests. The results indicated a strong pro‐apoptotic effect, triggering programmed cell death in cancer cells, an essential aspect of any effective cancer treatment. Additionally, the nanoparticles showed anti‐migratory behaviour, effectively inhibiting the cancer cells' ability to move and spread, thereby acting as a formidable tool against metastasis [62]. The work of Thapa's team shines for its innovation in dual‐action drug delivery and its potential to offer a more targeted and effective treatment strategy for the challenging condition of metastatic prostate cancer [62]. They show promise in diverse applications, from enhancing photothermal treatments to dual‐drug delivery systems for tackling complex cancer types. The research underscores the crucial role that nanotechnology can play in revolutionising cancer treatments.

### 17.12 Gold‐Deposited Zein Nanoshells

The research by Chauhan and associates aimed to harness the unique optical properties of gold for photothermal therapy, a treatment modality that uses light to generate localised heat to kill cancer cells [59]. Photothermal therapy has often been limited by the lack of biocompatible materials that also possess excellent photothermal properties. In this context, the team ingeniously deposited a layer of gold onto zein nanoparticles to create nanoshells. The gold layer contributed highly efficient light‐to‐heat conversion, thereby boosting the photothermal properties of the nanoparticles. Zein, a naturally occurring protein, served as the core of these nanoshells, ensuring their biocompatibility. The researchers utilised various characterisation techniques such as spectroscopy and electron microscopy to assess the morphological and photothermal attributes of these nanoshells. Additionally, in vitro experiments revealed that these gold‐deposited zein nanoshells could efficiently generate enough heat upon light exposure to induce cancer cell death, thereby affirming their potential for photothermal cancer therapy [59] (Figure 6).

### 17.13 Breast Cancer Treatment With Micelles

Sabra and her team took an alternative route by designing amphiphilic zein‐lactoferrin micelles to tackle breast cancer [63]. The micelles were engineered for the co‐delivery of two distinct drugs, rapamycin and wogonin, aimed at a synergistic therapeutic effect. Rapamycin is known for its immunosuppressive and anti‐proliferative properties, while wogonin is an active flavonoid with various pharmacological activities, including anti‐tumour effects. The micelles demonstrated remarkable stability over an extended period, a key attribute for their in vivo application. Furthermore, they significantly curtailed angiogenesis, the process of new blood vessel formation that often supplies tumours with nutrients. This angiogenic inhibition essentially starves the tumour, aiding in its demise [63]. Tests involving cell cultures and animal models showed the micelles' remarkable dual‐action capabilities. Not only did they facilitate the targeted delivery of both rapamycin and wogonin, but they also enhanced the overall therapeutic efficacy by reducing angiogenesis [63].

### 17.14 Sodium Deoxycholate‐Packed Paclitaxel

In a pioneering endeavour, Gagliardi and colleagues embarked on a groundbreaking strategy, harnessing the potential of sodium deoxycholate to encapsulate paclitaxel within zein nanoparticles [64, 65]. Recognising the challenge of paclitaxel's non‐specific cytotoxicity, their innovative approach sought to enhance its therapeutic effectiveness while mitigating adverse effects. Remarkably, their formulation not only preserved the structural integrity of the nanoparticles but also demonstrated a notable improvement in the entrapment efficiency of the drug. This milestone contribution holds significant promise in the realm of cancer therapeutics, offering a novel avenue for optimising paclitaxel's efficacy with reduced collateral damage [64, 65].

### 17.15 Treatment of Lung Cancer

Yu and his research team undertook a meticulous exploration targeting A549 lung cancer cells, utilising zein nanoparticles to encapsulate the potent cytotoxic agent, Maytansine [14]. This astute endeavour bore fruit in the form of enhanced cellular uptake of the encapsulated agent, outperforming free Maytansine. Such heightened cellular internalisation directly translated to elevated anti‐cancer efficacy, positioning zein nanoparticles as a potential enhancer of lung cancer treatment strategies. This accomplishment underscores the role of nanocarriers in augmenting drug delivery and effectiveness in cancer therapy [44, 66].

### 17.16 Disulfide‐Linked Paclitaxel

Hou and colleagues embarked on a sophisticated endeavour involving the engineering of a multifaceted prodrug—a fusion of paclitaxel, zein and a disulfide linker [67]. By capitalising on the unique reductive microenvironment within cancer cells, this innovative approach offered targeted drug release. The culmination of this research unveiled compelling results—a marked reduction in tumour size. This seminal contribution underscores the potential of disulfide‐linked prodrugs as an avenue for refining cancer treatment paradigms, providing a glimpse into the future of precision therapeutics [67].

### 17.17 Breast Cancer and Beta Carotene

Jain and his associates delved into the intricate landscape of breast cancer treatment, employing zein nanoparticles as vehicles for delivering beta‐carotene [45, 68]. Their comprehensive investigation yielded a striking revelation—the nanoparticle‐mediated delivery system engendered superior pharmacokinetic profiles compared to conventional administration. This advancement carries profound implications, potentially revolutionising the therapeutic approach to breast cancer by optimising drug delivery efficiency and elevating treatment outcomes [45, 68].

### 17.18 Resveratrol‐Loaded Zein Nanoparticles

Scientists embarked on a quest to harness the potent properties of resveratrol, an antioxidant compound with promising anticancer effects, through encapsulation within zein nanoparticles [69]. The consequential findings revealed heightened antioxidant activity and potentiated anticancer effects, surpassing the capabilities of free resveratrol. This innovative strategy showcases the potential of zein nanoparticles to amplify the therapeutic impact of natural agents, presenting a convergence of nanotechnology and medical science with profound implications for cancer treatment [69].

### 17.19 Treatment of HepG2 Cells

Alhakamy and his team directed their focus towards the treatment of HepG2 liver cancer cells, employing zein nanoparticles laden with Lovastatin [70]. The intricate exploration yielded promising outcomes, including apoptosis induction and robust anti‐proliferative activity. These findings resonate strongly with the quest for effective liver cancer treatment strategies, positioning zein nanoparticle formulations as a potent contender in the quest to revolutionise therapeutic approaches in hepatocellular carcinoma [70].

### 17.20 Curcumin Delivery Targeting CD44

Scientists embarked on an intricate endeavour involving zein nanoparticles cross‐linked with hyaluronic acid, serving as carriers for the precise delivery of curcumin into cancer cells [71]. Their comprehensive study unveiled a significant achievement—remarkable compatibility with cellular and hemodynamic environments. This sophisticated approach holds transformative potential, hinting at the future of targeted cancer therapies that capitalise on the interplay between advanced nanocarrier systems and the intricacies of cancer biology [71].

### 17.21 Paclitaxel and PEGylated Zein

Scientists navigated the complex terrain of PEGylated zein nanoparticles for paclitaxel delivery, amalgamating enhanced stability with the advantages of targeted drug release [72]. Through PEGylation, the nanoparticles achieved improved stability and solubility, while zein's core facilitated specific drug delivery. The in vitro evaluations yielded promising results—heightened cytotoxicity, underscoring the potential of this formulation to serve as a cornerstone in precision cancer treatments. This sophisticated approach encapsulates the spirit of precision medicine, poised to revolutionise the therapeutic landscape [72].

### 17.22 Photocytotoxicity of Indocyanine Green

Lee and his research group worked on zein phosphatidylcholine hybrid nanoparticles loaded with Indocyanine green [73]. This configuration was shown to stabilise the Indocyanine green, thus enhancing its phototoxicity, which makes the substance more effective in light‐based cancer therapy [73]. Zein nanoparticles offer a versatile and potentially groundbreaking platform for enhancing the delivery and effectiveness of anticancer drugs across various types of cancers. Their tailorability, biocompatibility and demonstrated ability to enhance drug efficacy suggest that zein nanoparticles may play a significant role in shaping future cancer treatment paradigms.

## 18 Unlocking the Power of Zein: Comprehensive Analysis of Anticancer Drug Administration Routes

Zein's versatility and inherent benefits (biocompatibility, biodegradability, amphiphilicity, self‐assembly and modifiability) make it a highly promising material for developing next‐generation anticancer drug delivery systems. By tailoring zein‐based formulations for specific administration routes, researchers aim to improve drug stability, increase bioavailability, achieve targeted delivery, control drug release and ultimately enhance the efficacy of chemotherapy while minimising its severe side effects, thus unlocking new possibilities for more effective and personalised cancer treatments (Table 4).

**TABLE 4:** Comprehensive analysis of anticancer drug administration routes Leveraging zein.

Administration route	Key insights and challenges	Role and potential of 005Aein
Intravenous injection	Direct pathway to systemic circulation. High bioavailability rate. Risk of adverse immune reactions. Concerns with drug solubility.	Biocompatible due to maize origin. Enhances solubility of anticancer agents. Ensures stable circulation.
Oral administration	Preferred for ease and convenience. Challenges with stomach acidity, digestive enzymes, and intestinal barriers.	Structural properties ensure drug protection. Controlled release safeguarded from the gastrointestinal tract.
Transdermal innovations	Enhanced delivery over traditional patches. Painless penetration of skin's outer layer.	Robust structural integrity. Sustained drug release.
Inter tumoural injections	Direct tumour targeting. Tumour microenvironment challenges.	In situ gel formulation with zein ensures drug retention. Optimised drug availability.
Pulmonary inhalation	Swift therapeutic onset for localised lung diseases. Lung defence mechanisms pose delivery challenges.	Zein nanoparticles bypass lung defences. Protective encapsulation ensures optimised delivery.

### 18.1 Intravenous Injection: The Direct Gateway With Complexity

The intravenous (IV) route, often deemed the most direct pathway to systemic circulation, boasts an unparalleled bioavailability rate, ensuring drugs quickly reach their target sites. However, the double‐edged sword of IV administration is its susceptibility to potential adverse reactions. High purity levels are paramount, as contaminants can trigger severe immune responses. Furthermore, repeated IV administrations can challenge patient comfort and compliance. An emerging concern in IV drug delivery pertains to anticancer agents with poor water solubility, posing the risk of precipitate formation and rapid systemic clearance, thereby undermining therapeutic outcomes [74]. Zein's potential is game‐changing in this scenario. Its origin from maize makes it biocompatible, reducing adverse immune reactions. Leveraging zein nanoparticles can enhance the solubility of otherwise insoluble anticancer agents, ensuring stable circulation and optimal therapeutic delivery [75, 76].

### 18.2 Oral Administration: The Age‐Old Method With Modern Challenges

Swallowing a pill or capsule is an age‐old method that is preferred for its ease and convenience. But the journey of a drug from the mouth to systemic circulation is fraught with challenges [77]. Stomach acidity, digestive enzymes, and intestinal barriers often degrade or impede drug absorption. Zein steps in as a guardian shield. Its structural properties form a protective encasement, ensuring the drug's controlled release while safeguarding it from the gastrointestinal tract's hostile environment [78]. A seminal study by Shinde et al. [79] proved this concept by successfully delivering luteolin—a poorly soluble compound, via zein nanoparticles, resulting in enhanced absorption and therapeutic efficacy.

### 18.3 Transdermal Innovations: Pioneering Beyond Patches

The skin, while a protective barrier, also presents an opportunity for drug delivery, especially when oral or IV routes pose challenges [80]. Microneedles, minuscule projections that can painlessly penetrate the skin's outer layer, are gaining traction for their enhanced delivery over traditional patches [81]. Zein, with its robust structural integrity, is proving pivotal in this realm. A groundbreaking research [82] employed zein in crafting microneedles, demonstrating not only structural resilience but also the ability to sustain drug release over extended periods.

### 18.4 Intertumoural Injections: Precise Targeting Amidst Hostile Environments

Directly targeting the tumour site is a strategy gaining attention in oncology. But this is not without challenges: the tumour's microenvironment can degrade or expel drugs, limiting therapeutic efficacy [59, 61, 62, 63, 65, 68, 81, 83]. In situ gels, which transition from liquid to gel upon injection, offer the advantage of local retention. Xiaoying Cao's avant‐garde approach [84] integrated zein to formulate an in situ gel, which, when loaded with the potent drug doxorubicin, showed sustained release, optimising drug availability within the hostile tumour milieu.

### 18.5 Pulmonary Inhalation: Advancing Beyond Traditional Respiratory Therapies

Directly administering drugs to the lungs is advantageous, particularly for localised lung diseases, ensuring swift therapeutic onset [59]. Yet, the lung's defences, including mucus barriers and macrophages, can reduce drug retention. Zein nanoparticles have emerged as saviours in this space. Their protective encapsulation facilitates drug delivery past these defences. Fatima Hameedat's studies [13] delved into the promise of zein nanoparticles for insulin delivery, while Kamel et al. [66] further extended these findings, showcasing the nanoparticles' potential in optimising delivery of anticancer agents to lung tissues.

As pharmaceutical science continues to evolve, zein's multifaceted role in enhancing drug delivery across diverse administration routes underscores its importance in the future of therapeutics.

## 19 Advanced Functionalization and In‐Depth Exploration of Nanomedicine Delivery and the Rising Prominence of Zein‐Based Systems

The domain of nanomedicine has made monumental strides, especially when considering drug delivery to complex sites like tumours. Historically, the Enhanced Permeability and Retention (EPR) effect has been at the forefront of these efforts. The EPR effect capitalises on the inherent properties of tumour vasculature, where the larger gaps between endothelial cells in tumour blood vessels, coupled with the reduced ability of tumours to clear out substances due to a lack of effective lymphatic drainage, enable nanoparticles to accumulate preferentially in tumour tissues. This passive targeting mechanism, though a groundbreaking discovery in its time, has shown a set of intrinsic limitations in recent advanced applications. One major impediment of the EPR‐based delivery method is the system's inability to prevent the premature expulsion of nanodrugs from the bloodstream. This is largely because the body's sophisticated defence mechanism, primarily the mononuclear phagocyte system (MPS), is adept at detecting, capturing, and subsequently eliminating foreign particles. Such an expedited removal mechanism prevents the therapeutic agents from lingering in the bloodstream long enough, thereby limiting their chances to accumulate in the targeted tumour regions adequately. Moreover, the EPR effect does not guarantee a uniform spread of the nanodrugs within the tumour. Certain areas might receive a higher concentration of the drug, while others may be deprived. This differential distribution can potentially create therapeutic dead zones, allowing pockets of cancer cells to remain untreated. Over time, these residual cells can multiply, possibly leading to drug resistance and compromising the overall treatment's success rate. It is within this backdrop that researchers have been fervently exploring other promising strategies to overcome the shortcomings of EPR‐centric delivery. One of the standout contenders in this research race has been zein. This protein, predominantly extracted from maize, boasts a molecular structure rife with free carboxyl and amino groups. Such a structural configuration allows for multifaceted chemical interactions, enabling a myriad of drug conjugation possibilities and surface modifications. Furthermore, zein's credentials as a biocompatible and biodegradable material make it a particularly attractive option. Its ability to degrade naturally within the body, coupled with its low toxicity profile, ensures minimal side effects, thereby improving the patient's overall therapeutic experience.

In the context of its adaptability, zein's structure is amenable to a wide range of modifications. This means that scientists can potentially tailor zein‐based nanoparticles to address specific tumour characteristics or to respond to particular environmental triggers within the tumour microenvironment. Such precision targeting can drastically improve drug delivery efficiency while minimising collateral damage to healthy cells. Given this spectrum of advantages and the ongoing innovative research into zein's potential applications, it is becoming increasingly evident that zein‐infused nanoparticle delivery systems may well pave the way for the next big leap in cancer therapeutics and beyond.

## 20 Advancing Nano‐Drug Delivery Through Ligand‐Enhanced Targeting Utilising Zein as a Substrate

The realm of nanomedicine has historically leaned on the Enhanced Permeability and Retention (EPR) effect as its linchpin for nano‐drug delivery, specifically targeting tumour cells. However, as promising as the EPR effect has been, it has presented limitations concerning specificity, precision and efficiency. To address these challenges, the scientific fraternity is embracing a paradigm shift that seeks to optimise the nanoparticle surface to resonate more harmoniously with the tumour milieu. Ligand‐enhanced targeting, an innovative frontier in nanomedicine, hinges on the strategic coupling of specific ligands to the nanoparticle surface. These ligands are not arbitrarily chosen; they are meticulously selected based on their inherent capability to pinpoint and bind with impeccable specificity to receptors that are predominantly, if not exclusively, manifest on tumour cells. Such precise targeting is envisioned to significantly minimise deleterious off‐target effects, thereby augmenting the therapeutic index of the drug being delivered (Table 5).

**TABLE 5:** Receptor affinities, conjugation methodologies, implicated drugs and projected therapeutic outcomes.

Ligand	Target receptor	Receptor function	Binding mechanism to zein	Transported drugs	Therapeutic efficacy
Hyaluronic acid	CD44	Cell adhesion and migration	Carbodiimide reaction	Drug XYZ	High
Chondroitin sulphate	CD54	Inflammation & Immunity	Ionic hydrogen bonding	Drug ABC	Moderate
Folic acid	FRα	Vitamin uptake and metabolism	Carbodiimide reaction	Drug LMN	Very high
Biotin	Streptavidin	Biotin‐protein binding	Ionic hydrogen bonding	Drug DEF	High

Zein, a corn‐derived protein, offers immense potential in this ligand attachment paradigm. Its molecular structure, which is inherently replete with free carboxyl and amino functional groups, makes it a prime candidate for ligand tethering. As research has advanced, a suite of ligands—such as hyaluronic acid, chondroitin sulphate [63], folic acid [63] and biotin [63]—have undergone rigorous scrutiny for their applicability. Conjugating these ligands to zein, through methods like the carbodiimide reaction or ionic hydrogen bonding, not only enhances their stability but also optimises their tumour‐homing capabilities.

## 21 Delving Deeper Into Zein Nanoparticles: Stimuli‐Responsive Release and the Future of Precision Oncology

Historically, zein‐based nanoparticles have been at the forefront of drug delivery innovations, offering unparalleled advantages in the controlled release of encapsulated therapeutics. Multiple studies have corroborated their inherent dual‐phase release pattern [46], wherein the drug undergoes an immediate and rapid release, ensuring swift therapeutic intervention and is succeeded by a steady, extended release. This sustained phase is pivotal, ensuring the consistent presence of therapeutic concentrations within systemic circulation, thereby improving patient compliance and therapeutic outcomes.

Yet, as the pharmaceutical landscape continually evolves, a more nuanced approach has emerged: stimuli‐responsive zein nanoparticles. Every tumour microenvironment is endowed with certain unmistakable attributes—notably, an acidic pH balance and elevated concentrations of glutathione (GSH). Harnessing these distinct attributes has opened a new frontier in precision drug delivery. By meticulously engineering nanoparticles to respond specifically to these tumour‐centric conditions, the horizon of drug delivery stands to be redefined. Such hyper‐targeted drug release is not just about increasing therapeutic efficacy; it is equally about mitigating collateral damage to healthy tissues, thereby dramatically reducing potential side‐effects [85] (Table 6).

**TABLE 6:** Delineating the underlying mechanisms of response, the specific triggers presented by the tumour milieu, the consequential release characteristics, and the broader ramifications for precision drug delivery.

Responsive mechanism	Tumour microenvironmental trigger	Release characteristic	Therapeutic implication
pH‐sensitive modulation	Predominantly acidic environment (pH < 7.4)	Release acceleration under acidic conditions	Ensures drug localization and concentrated action within tumour milieu, curtailing non‐specific cellular damage
GSH‐induced release	Elevated intratumoural GSH concentrations	Prompt release in high GSH conditions	Exploits the tumour's high GSH levels for precise intracellular drug delivery, optimising therapeutic window
Combined pH/GSH‐sensitive mechanism	Interplay of acidic pH and elevated GSH	Modulated release catering to both environmental cues	Orchestrates a tailored drug delivery profile, optimising therapeutic concentrations across diverse tumour microenvironments

Given this paradigm shift brought about by stimuli‐responsive nanoparticles, oncological drug delivery is poised for transformative advancements. It's a transition from a ‘one‐size‐fits‐all’ approach to a highly tailored and responsive delivery mechanism, holding immense promise for personalised medicine and improved patient prognoses.

## 22 Delving Into pH‐Driven Strategies: The Multifaceted Nuances of Tumour Microenvironments

The intrinsic nature of tumours to maintain an acidic microenvironment has been a focal point of oncological research for decades. Tumours frequently manifest a unique acidic terrain, predominantly attributed to intricate biological processes like acidosis and cellular hypoxia [46]. This acidic predisposition offers a stark contrast to the relatively neutral pH observed in regular, healthy tissues [85]. Such inherent characteristics of tumours have paved the way for designing specialised drug delivery systems, accentuating the possibility of highly targeted therapeutic interventions [86].

At the heart of this strategic endeavour lies the potential of metal–ligand coordination bonds. Their notable sensitivity to pH changes, even subtle ones, enables them to act as efficient tools in drug delivery mechanisms [87]. Within this framework, tannic acid has risen to prominence due to its exemplary metal‐chelating properties. Recent advancements suggest that tannic acid can be efficiently employed to construct innovative, pH‐responsive coatings on zein nanoparticles [88]. It meticulously maps out the far‐reaching implications of PDA‐coated zein nanoparticles, unveiling their capability to elicit distinct pH‐sensitive drug release behaviours. What is truly groundbreaking, however, is the burgeoning research that synergises the precision of pH sensitivity with the specificities of receptor targeting [89]. Hongdi Wang's illustrious research offers a deep dive into this combined approach [90]. Wang's novel methodology synthesises zein nanoparticles infused with a dual‐function mechanism: one that is simultaneously oriented towards precise receptor targeting and attuned for pH‐responsive release (Table 7). This integrated approach promises to redefine therapeutic paradigms.

**TABLE 7:** A deep dive into pH‐sensitive strategies for zein nanoparticles.

Strategic approach	Principal component	Underlying mechanism	Lead researcher	Significant outcomes and inferences
Metal–ligand coordination mechanism	Tannic acid	Dynamic drug release modulated by pH gradients	Hongshan Liang	Unearthed the potential of tannic acid as a pH‐sensitive coating, optimising drug release profiles
Biopolymer‐driven sensitivity	Polydopamine (PDA)	Activation in response to tumour‐specific acidic conditions	Liqiong Zha	Validated the promise of PDA‐coated nanoparticles for achieving precise drug delivery
Integrated dual‐function paradigm	Confluence of receptor targeting and pH‐sensitivity	Hybrid precision targeting with dynamic release	Hongdi Wang	Introduced a novel approach amalgamating receptor precision with pH‐responsiveness, hinting at future therapeutic directions

In summation, by intricately understanding and harnessing the distinctive pH characteristics of tumour microenvironments, the biomedical community stands on the precipice of a significant breakthrough in oncological drug delivery. As we continue our journey, the eventual goal remains clear: pioneering treatments that are meticulously tailored to the unique attributes of each tumour, ensuring enhanced therapeutic efficacy and patient outcomes.

## 23 Optimising GSH‐Concentrated Environments in Advanced Nano‐Drug Delivery

The tumour microenvironment stands out in oncological research because of its specific biochemical properties. A significant deviation from typical body fluids is the elevated levels of reduced glutathione (GSH) in these tumour sites. These concentrations can be anywhere from 100 to 1000 times more than what is found in standard body fluids such as blood and extracellular liquid [91]. Moreover, the hypoxic conditions prevailing in tumour tissues serve to compound this GSH concentration, making them about four times higher than their counterparts in normal tissues [92]. Several trailblazing research initiatives have been undertaken to devise nano‐drug delivery systems tailored for these GSH‐enriched environments. A predominant strategy being explored involves the utilisation of disulfide bonds. These bonds possess a remarkable sensitivity to GSH, breaking apart and thereby releasing their linked drugs or attached carrier molecules, which contain functional groups like ‐NH2, ‐OH or ‐COOH through processes such as amide or esterification [67]. The eventual outcome of this innovative approach is the formation of nanoparticles that can self‐assemble in response to GSH levels. Zein, a renowned protein celebrated for its dual nature of being both hydrophilic and hydrophobic (amphiphilic), has shown to play a pivotal role in these formulations (Table 8). It is not just its amphiphilic nature, but also its amenability to chemical modifications that positions zein as a key player. When transformed into nanoparticles, zein can amplify the in vivo circulation duration of drugs by an impressive factor of approximately 7.2 [93].

**TABLE 8:** Detailed insights into zein‐based GSH‐responsive nanoparticles.

Parameter	Detailed description and insights
Chemical structure	Zein and paclitaxel (PTX) are connected via esterification, creating disulfide bonds sensitive to GSH.
Reaction environments explored	Neutral pH (7.4) without GSH and buffer conditions with 10 mM GSH.
PTX Release dynamics (immediate)	The nanoparticles are essentially dormant under standard conditions but display rapid adaptability when exposed to GSH, releasing up to 90% of the PTX within an initial 5‐min interval.
PTX release window (2‐h period)	In environments mimicking tumour GSH concentrations, there's a progressive release, culminating in a 95% discharge rate in 2 h.
Cell interaction profile	Exhibits dual properties: Potent anti‐cancer activity rivalling pure PTX and simultaneous non‐toxic interaction with NIH/3T3 fibroblast cells.
In vivo tumour response	Zein‐S‐S‐PTX_NP showcases unparalleled efficacy in live models, significantly dwarfing tumour sizes by up to 50%, demonstrating superiority over other tested compounds.
Nano‐particle formation mechanism	Zein's amphiphilic nature combined with its chemical modification capacity results in self‐assembling nanoparticles, ensuring enhanced drug circulation in vivo.
Therapeutic implications	Given their high specificity and rapid responsiveness, these nanoparticles promise to reduce systemic side effects, ensuring targeted tumour therapy.
Future prospects	The introduction of other responsive elements, possibly diversifying the range of environmental triggers beyond GSH, offers a vast horizon for exploration.

During experiments, these nanoparticles exhibited a unique adaptability. In conditions resembling normal body fluids (pH 7.4 without GSH), zein‐S‐S‐PTX_NP maintained a dormant state, ensuring no premature drug release. However, their responsiveness was brought to the fore when placed in a GSH‐rich buffer. Almost instantaneously, they released close to 90% of PTX within a mere 5 min, and reached a zenith of 95% in just 2 h. This ensures that in real‐world applications, these nanoparticles would only become active in tumour environments, exemplifying a superbly controlled drug delivery mechanism. When tested on cells, zein‐S‐S‐PTX_NP displayed anti‐cancer prowess equivalent to that of unmodified PTX and was found to be non‐toxic to NIH/3T3 cells (Table 8). Further studies in live models reaffirmed their effectiveness, with tumours treated with zein‐S‐S‐PTX_NP showing significant size reduction.

## 24 Magnetic Targeting in Tumour Therapeutics: Innovations With SPIONs and Molecular Precision

**FIGURE 7:**
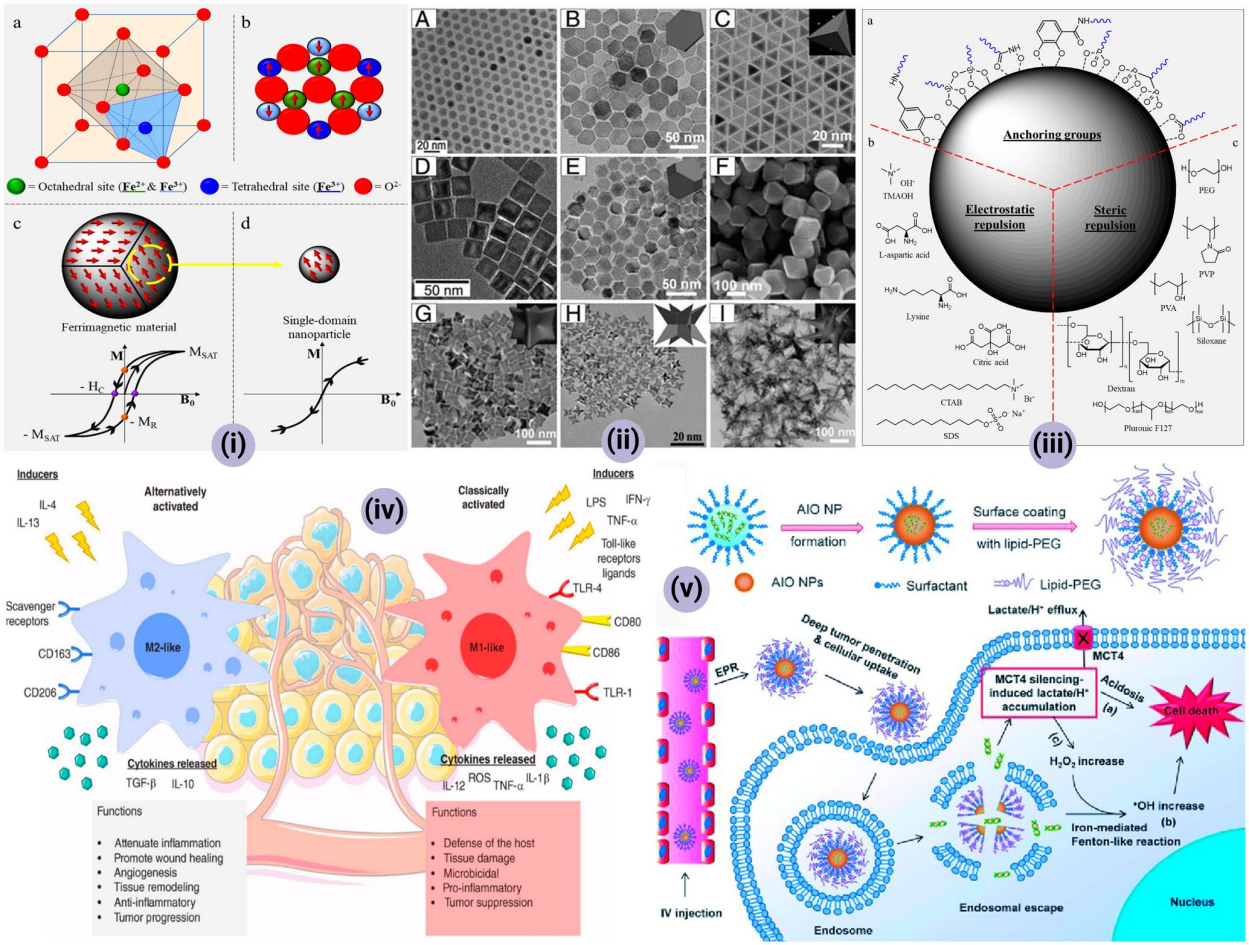
(I) (a) Illustration of the face‐centred cubic arrangement of O^2−^ anions and location of ferric and ferrous ions in octahedral and tetrahedral sites; (b) illustration of the ferrimagnetic network formed by ferrous and ferric ions in magnetite; (c) illustration of domain formation in ferrimagnetic material and resulting magnetisation behaviour; (d) illustration of a single‐domain nanoparticle and resulting superparamagnetic curve [94]; (II) Examples of particle morphologies: (A) nanospheres; (B) plates; (C) tetrahedrons; (D) cubes; (E) truncated octahedrons; (F) octahedrons; (G) concaves; (H) octapods; (I) multibranches. Reprinted from [95]; (III) (a) Anchoring groups grafted on an iron oxide surface, from left to right, dopamine, siloxane, hydroxyamate, 2,3‐dihydroxybenzamide, mono‐ and bis‐phosphonate, and carboxylate; (b) examples of capping agents providing electrostatic stabilisation; (c) polymer coatings providing steric stabilisation [94]; (IV) Binary categorisation of proinflammatory (M1‐like) and anti‐inflammatory (M2‐like) macrophages in the tumour microenvironment. Reprinted from [96]; (V) Illustration of (A) SPION‐based RNAi platforms (AIO: Amorphous iron oxide) and (B) their interaction with tumour cells. Upon i.v. administration, nanoparticles accumulate in tumour cells through the EPR effect. Internalisation of the nanoparticles within the endosome and subsequent release of iron ions leads to osmotic pressure and/or endosomal membrane oxidation. The resulting endosomal escape induces the release of RNAi and iron ions, resulting in MCT4 silencing and oxidative stress via the Fenton‐like reaction. Reprinted from [97].

Magnetic targeting in tumour therapeutics has evolved into an intricate and multidimensional approach, leveraging the precision of magnetism to navigate drug delivery. At the heart of this innovation lies the Superparamagnetic Iron Oxide Nanoparticles (SPIONs) (Figure 7). Recognised not only for their biocompatibility and ability to be synthesised with meticulous dimensions, SPIONs have gained significant credibility by being the singular magnetic nanomaterial to functionalize Zein which secured the endorsement of the US FDA for biomedical applications [36]. However, to unlock their optimal therapeutic potential, it is imperative to move beyond basic magnetic targeting. Researchers have discerned this necessity, integrating aptamers—a category of specialised molecules known to bind specific targets—onto carrier surfaces, thus amplifying drug uptake in tumours. For instance, one unique study melded this intricate philosophy with practical innovation. Using an elaborate assembly of chondroitin sulphate, sulfapyridine and the protein zein, they pioneered a unique amphiphilic structure encapsulating both SPIONs and the anti‐cancer compound celastrol (CST) [98]. This strategic construction ensures both magnetic and molecular precision in targeting. In a parallel vein, Some demonstrations and investigations took this a step further [98]. Scientists ingeniously coupled SPIONs with gefitinib (GEF) inside folic acid‐conjugated zein complexes. With folic acid being a well‐documented agent in enhancing tumour cell internalisation, these complexes, under an external magnetic directive, showcased superior anti‐proliferative efficacy (Figure 7). Additionally, Sabra's exploratory work underscores the versatility of these approaches [63]. By uniting lactoferrin—a protein targeting tumour cell surface receptors—with zein in amphiphilic fragments, and subsequently encapsulating SPIONs and the potent drug dasatinib, Sabra et al. laid down a blueprint for multifaceted tumour targeting, both magnetic and receptor‐specific (Table 9).

**TABLE 9:** Comparative analysis of pioneering studies on SPION‐embedded magnetic targeting approaches.

Core nanoparticle	Added components and drugs	Embedded strategy	Core findings and outcomes
Zein‐based nanoparticles	Chondroitin sulphate, sulfapyridine, CST, SPIONs	Dual‐layered approach integrating chondroitin sulphate molecular targeting with magnetic orientation.	Achieved heightened cellular uptake, paving the way for intensified anti‐tumour activity.
Folic acid‐conjugated zein	GEF, SPIONs	Fusion of magnet‐guided targeting & folic acid conjugation, maximising drug delivery efficiency.	Demonstrated superior cellular absorption; remarkable anti‐proliferative action against A549 cells.
Lactoferrin‐zein hybrid fragments	Dasatinib, SPIONs	A composite mechanism, blending lactoferrin receptor targeting on tumour cells with magnetic navigation.	Established a benchmark in tumour‐specific delivery with pronounced uptake in tumour cells, ensuring minimised collateral damage.

## 25 Advancements in Gel Encapsulation: Harnessing Zein's Potentials and Improving Nanoparticle Stability

The field of drug delivery has witnessed zein emerge as a prime contender owing to its inherent hydrophobic properties, biodegradability and biocompatibility. These attributes enable zein‐derived nanoparticles to considerably elevate the water solubility of incorporated drugs. Yet, a meticulous evaluation indicates certain areas, including the aqueous solubility of its surface layer, overall stability and drug release trajectory, where advancements are still coveted [80].

In the quest to optimise these properties, hydrogels, defined by their unique three‐dimensional polymeric configurations, have been spotlighted. These frameworks can retain significant water quantities due to their intertwined polymer chains. By integrating zein‐based, drug‐loaded nanoparticles within such hydrogel systems, there is potential to harness the hydrophilicity of hydrogels (Table 10). This synergy may curtail the tendency of nanoparticles to be prematurely eliminated by macrophages in the bloodstream. Additionally, hydrogels bring to the table their innate porous nature and propensity to swell, attributes that might further finetune the controlled drug release from the encased nanoparticles [57].

**TABLE 10:** Enhanced gel‐encased zein nanoparticle delivery mechanisms.

Parameters	Description	Key findings
Material composition	Zein (hydrophobic, biodegradable, biocompatible) Hydrogel (3D polymeric configurations)	Enhances water solubility of drugs
Zein's special attributes	Isoelectric point: 6.2 Hydrophobicity Biocompatibility	Suitable for drug encapsulation
Hydrogel's role	Retention of water Porosity and swelling property Prevents premature elimination by macrophages	Augments drug release control
Research case study	Study by Priyanka Kaushik [57]	Pectin hydrogel‐encapsulated doxorubicin‐loaded zein nanoparticles
Innovation basis	Zein's isoelectric point vs. Pectin's polyanionic character at pH = 2.47	Strong electrostatic interactions
Drug charge dynamics	Drug exhibits a positive charge, enhancing uptake by negatively charged HeLa cells	Enhanced cellular drug uptake
Drug release pattern	Initial rapid release followed by extended controlled release	Extended therapeutic window
Therapeutic agent	Doxorubicin	Triggers mitochondrial‐dependent apoptosis
Cellular response	Selective toxicity towards HeLa cells while sparing HEK293 cells	Targeted therapeutic efficacy

A noteworthy endeavour in this domain is the research spearheaded by Priyanka Kaushik [57]. Kaushik's team ingeniously encapsulated doxorubicin‐loaded zein nanoparticles within pectin hydrogels without necessitating any external cross‐linking agents. The basis of this innovation lies in the distinct isoelectric point of zein, pegged at 6.2, juxtaposed with pectin's polyanionic character. At a pH environment of 2.47, a potent electrostatic interplay between these two biopolymers was observed. Empirical evidence from Kaushik's study underscores that pectin demonstrated a preferential affinity towards DOX‐infused zein nanoparticles, majorly driven by these electrostatic interactions. Another salient discovery was the overall positive charge of the drug entity, which when combined with the negative charge inherent to HeLa cell surfaces, augments the cellular drug uptake. This optimisation potentially catapults the therapeutic efficacy of the drug.

The drug release dynamics, when assessed in vitro with the introduction of DOX into the zein‐Pectin (ZP) hydrogel matrix, revealed a biphasic pattern: an upfront rapid release superseded by a modulated, extended release. The rhythm of this drug dispersal is instrumental in shaping the therapeutic index of anti‐cancer regimens. A consistent, protracted release ensures a sustained therapeutic window. It is also pivotal to highlight that doxorubicin, an established anti‐cancer molecule, mediates its curative prowess by triggering mitochondrial‐dependent apoptosis. Intriguingly, the study's findings shed light on a discerning toxicity profile, with a marked preference towards HeLa cells, while largely sparing HEK293 cells. The Table 9 offers a comprehensive overview of the key characteristics and research findings related to gel‐encased zein nanoparticle delivery mechanisms. It showcases the unique attributes of both zein and hydrogels, the research foundations, and the resultant therapeutic implications.

In essence, the adaptability and multifunctionality of zein offer a promising future for refining drug delivery systems. By marrying the innate properties of zein with cutting‐edge scientific advancements, the realm of cancer therapeutics may be on the brink of a revolution. Zein‐based nanoparticles have emerged as a potent tool in the realm of drug delivery, underlined by their hydrophobicity, biocompatibility and biodegradability. Their dual‐phase release patterns and stimuli‐responsive behaviours make them particularly suited for targeted drug delivery, especially in oncology. Various methodologies have been developed to enhance their utility:

pH Sensitivity: Given the inherently acidic environment of tumour sites, scientists have innovated with metal–ligand coordination bonds, tannic acid and polydopamine (PDA) to foster pH‐responsive drug delivery mechanisms. GSH‐Responsive Systems: The heightened levels of GSH in tumour microenvironments have inspired the development of nanoparticles that respond to these specific concentrations, ensuring more precise drug release. Magnetic Guidance: The infusion of magnetic nanoparticles, notably SPIONs, provides a mechanism to guide drug‐loaded nanoparticles directly to tumour sites using external magnetic fields. Hydrogel Integration: Merging zein's natural properties with the benefits of hydrogels enhances nanoparticle stability, improves water solubility and fine‐tunes controlled drug release properties.

## 26 Recent Studies on Zein Nanoparticle Based Cancer Therapy

Recent studies emphasise the therapeutic potential of Zein–sodium caseinate–diosmin nanoparticles (ZCD‐NPs) against ovarian cancer [99]. Scientists have demonstrated their capacity to significantly enhance diosmin's bioavailability, selectively induce cancer cell death through cytotoxic and apoptotic mechanisms, and concurrently exhibit potent antioxidant properties that augment their overall efficacy [99]. To further elucidate the intricate mechanisms of mitochondrial‐mediated apoptosis, future investigations incorporating analyses of reactive oxygen species (ROS) and mitochondrial membrane potential (MMP) should be performed. Quantifying ROS can illuminate the role of oxidative stress in cancer cell death, while tracking MMP changes will provide important insights into mitochondrial dysfunction, a critical event in the apoptotic cascade. Another study focused on loading zein nanoparticles with tyrosine kinase inhibitors (TKIs). The nanoparticle system was developed to deliver the TKIs, specifically erlotinib (Er‐GA‐ZNPs) and gefitinib (Gef‐GA‐ZNPs), which exhibited excellent encapsulation efficiency [100]. These TKI‐loaded zein nanoparticles markedly decreased the IC50 values of the free TKIs across three different cancer cell lines, indicating enhanced efficacy [100]. Mechanistically, treatment with these nanoparticles led to increased generation of ROS and depolarisation of the mitochondrial membrane in the treated cancer cells, which are important indicators of mitochondrial‐mediated apoptosis similar to the previous experiment [100]. Furthermore, the nanoparticles effectively suppressed cell migration, suggesting a reduction in the metastatic potential of the cancer cells [100].

## 27 Challenges and Outcomes

In the innovative drug delivery arena, the adoption of zein presents unique complexities. A defining trait of zein, its hydrophobic nature, is a double‐edged sword. While it facilitates the solubility of hydrophobic drugs, it simultaneously poses stability issues for the resulting formulations. This inherent trait gets further complicated by zein's alcohol solubility; the necessary use of alcohol in its production might leave potentially hazardous residues. Zein nanoparticles, being protein‐based, can face stability issues related to aggregation, denaturation, and microbial contamination during long‐term storage. Stability is highly dependent on the formulation conditions, including pH, temperature, storage medium and the presence of stabilisers. Coating zein nanoparticles with hydrophilic polymers like PEG or polysaccharides (e.g., chitosan) can create a steric barrier, preventing aggregation and improving colloidal stability in aqueous media. Furthermore, its rapid clearance from the body and its plant‐based origin raise concerns about its therapeutic efficacy and potential unpredicted immunogenic reactions. Shifting the focus from zein, another significant challenge arises in nanomedicine for lung cancer treatment, particularly when relying on cell lines for efficacy evaluation. These cell lines can be unrepresentative of certain lung cancer subtypes and lack the intricacies of living tumour tissues. The prevalent 2D cell models fail to replicate the in vivo cellular responses due to their inability to mirror the exact cell morphology and functionality. Moreover, the intricacies of cytotoxicity assays, pivotal for safety evaluations, get entangled with various unpredictable interactions, especially when high concentrations of nanoparticles are involved.

Strategies such as cross‐linking with anionic materials, including alginate and hyaluronic acid, have been put forth to enhance the stability of zein‐based formulations. Integrating surfactants like pluronic, tween and lecithin could further augment the solubility and stability. Ensuring a meticulous ethanol volatilisation process during zein production can alleviate concerns regarding alcohol residues. Encapsulation techniques, like housing zein in water‐soluble gels or employing PEG modifications, mitigate its rapid elimination issues. Expanding the breadth of research, particularly involving a broader spectrum of organisms, is crucial to ensure clinical safety. In nanomedicine research for lung cancer, the advent of 3D cell models shows promise. These models could bridge in vitro and in vivo studies, bringing research closer to actual physiological responses. Additionally, enhancing the protocols for cytotoxicity assays, optimising nanoparticle concentrations and establishing a robust system of baseline quality controls can pave the way for more accurate and reliable results.

Some studies have indicated that zein can elicit an immune response, including the production of anti‐zein antibodies. This response is generally reported to be mild. The long‐term impact of this immune response, especially with repeated administration, needs further investigation. It is crucial to assess if chronic exposure could lead to hypersensitivity reactions or other adverse immunological effects in a subset of patients. Surface modifications of ZNPs, intended for targeting or stability, could also influence their immunogenicity. These modifications need to be thoroughly evaluated for their potential to trigger immune responses over extended periods. Zein is biodegradable, but the rate of degradation and the fate of its breakdown products in the body over long periods are important to understand. Slow degradation could lead to accumulation in certain tissues or organs. The clearance mechanisms of ZNPs from the body following long‐term administration need to be elucidated. Impaired clearance could also contribute to potential toxicity. In a biological environment, nanoparticles are rapidly coated by a layer of biomolecules, primarily proteins, forming a ‘protein corona’. This corona can influence the nanoparticle's size, surface charge, cellular uptake, biodistribution, and interaction with the immune system. The composition of the protein corona can change over time and vary between individuals, potentially affecting the long‐term fate and toxicity of ZNPs. Long‐term exposure to ZNPs, even if initially well‐tolerated, could potentially lead to chronic toxicity in specific organs or tissues. Studies with extended administration periods in animal models are necessary to assess this risk. Factors such as nanoparticle size, surface charge, and the presence of encapsulated drugs can influence chronic toxicity profiles. Immune responses and toxicity can vary significantly between animal models and humans. Therefore, careful translation of preclinical long‐term safety data to clinical settings is essential. Long‐term studies focusing on the kinetics of immune responses to ZNPs, including the type of antibodies produced and the activation of immune cells over extended periods, are crucial. Detailed investigation of the degradation pathways of zein in vivo and the potential for accumulation of ZNP components or their degradation products in different organs after chronic exposure is needed. Understanding how the protein corona evolves over time in vivo and its impact on ZNP interactions with the immune system and target cells during long‐term therapy is vital. Chronic toxicity studies in relevant animal models with long administration periods and thorough histopathological and biochemical analysis are necessary to identify potential long‐term adverse effects.

Bringing zein‐based formulations to the market faces several regulatory hurdles, common to nanomedicines and specific to the novelty of these formulations. Regulators require thorough physicochemical characterisation of nanoparticles, including size, size distribution (PDI), shape, surface charge (zeta potential), porosity and stability under various conditions. The complexity arises from the dynamic nature of nanoparticles in biological environments (e.g., protein corona formation), making it challenging to define and control their critical quality attributes consistently. Developing robust and reproducible analytical methods for characterising these complex systems is a significant hurdle. Evaluating the safety of nanomaterials, including potential immunogenicity, biodistribution, accumulation in organs and long‐term toxicity, requires sophisticated preclinical testing. The lack of standardised protocols for nanomaterial safety assessment can lead to increased regulatory scrutiny and the need for extensive data. Understanding the interaction of ZNPs with biological systems at the nanoscale is crucial for predicting and mitigating potential adverse effects. Developing scalable and reproducible manufacturing processes for ZNPs with consistent quality attributes is a significant challenge. Ensuring batch‐to‐batch consistency in terms of size, drug loading, release profiles and surface properties is critical for regulatory approval. The transition from laboratory‐scale synthesis to large‐scale industrial production often requires significant optimisation and validation. Demonstrating the added value of the nanoparticulate formulation (e.g., improved bioavailability, targeted delivery, enhanced efficacy) compared to conventional formulations is essential. Clinical trial design needs to account for the unique pharmacokinetic and pharmacodynamic properties of nanomedicines. Establishing a clear link between the nanoparticle characteristics and the observed clinical benefits can be complex. The regulatory landscape for nanomedicines is still evolving. Clear and specific guidelines for zein‐based nanotherapeutics might be lacking, leading to uncertainty for developers. Determining whether a zein‐based formulation is classified as a drug, biologic, or a combination product can influence the regulatory pathway. Navigating the requirements for novel excipients (zein in a nanoparticle form) can add complexity to the approval process. Regulators may require specific post‐market surveillance studies to monitor the long‐term safety and efficacy of nanomedicines in a larger patient population. Understanding the potential for rare or delayed adverse events associated with nanomaterials is crucial. Early and frequent engagement with regulatory agencies (e.g., FDA) is crucial to understand their expectations and address potential concerns proactively. Investing in robust analytical methods for comprehensive characterisation of ZNPs is essential for demonstrating consistent product quality. Conducting thorough preclinical studies, including detailed biodistribution, toxicology, and immunogenicity assessments, is necessary to build a strong safety profile. Focusing on demonstrating a clear clinical benefit and added value of the zein nanoparticle formulation compared to existing therapies is vital for approval. Developing well‐defined and scalable manufacturing processes with stringent quality control measures is critical for consistent product supply.

Differences in gastric pH, enzyme activity, gut motility and the composition of the gut microbiome can affect the stability of ZNPs, drug release kinetics and absorption. Variations in liver enzyme activity can influence the metabolism and clearance of both the drug and potentially the zein nanoparticles themselves. Differences in blood flow, protein binding capacity, and the presence of specific proteins in the blood can affect the biodistribution and cellular uptake of ZNPs. Baseline immune status and individual immune responses to the nanoparticles or the encapsulated drug can vary significantly, influencing the overall therapeutic outcome and potential for adverse effects. The stage and severity of the disease can affect the physiological environment at the target site (e.g., tumour microenvironment, inflammation levels), influencing nanoparticle penetration, drug release and cellular uptake. Genetic variations in drug metabolising enzymes, transporters, and target receptors can lead to differences in drug response and nanoparticle interactions. The presence of other health conditions can influence drug pharmacokinetics and pharmacodynamics, potentially affecting the effectiveness of ZNP‐based therapies. Dietary components can interact with nanoparticles in the GI tract, affecting their stability and drug release. Other drugs a patient is taking can interact with the ZNP formulation. Exposure to certain environmental factors can influence a patient's physiological state and potentially affect nanoparticle behaviour. For targeted ZNPs, the level of expression of the target receptor on patient cells can vary, influencing the extent of drug delivery to the intended site. Once taken up by cells, the intracellular trafficking pathways of ZNPs can differ between patients, affecting drug release and efficacy. Developing predictive biomarkers that can identify patients who are more likely to respond to specific ZNP‐based therapies would be highly valuable for personalised medicine approaches.

## 28 Future Perspectives

Tumour tissues often exhibit a lower extracellular pH compared to normal tissues. ZNPs can be engineered with pH‐sensitive materials or linkers that trigger drug release specifically in the acidic TME, minimising premature drug release in systemic circulation. This can be achieved by incorporating polymers or using pH‐cleavable bonds. Certain enzymes, such as matrix metalloproteinases (MMPs) and cathepsins, are overexpressed in the TME and play a role in tumour invasion and metastasis. ZNPs can be functionalised with peptide substrates that are specifically cleaved by these enzymes, leading to localised drug release within the tumour. The TME often has a higher reducing environment compared to normal tissues. ZNPs can be designed with disulphide bonds that are cleaved by the high concentration of glutathione in the TME, resulting in controlled drug release. Some tumours are hypoxic (low oxygen levels). ZNPs can be modified with hypoxia‐activated prodrugs or targeting ligands that are activated or have increased affinity under hypoxic conditions, allowing for selective drug delivery to these resistant tumour regions. The TME can present physical barriers like dense extracellular matrix (ECM) and high interstitial fluid pressure (IFP). ZNP size, shape and surface charge can be optimised to enhance penetration through the ECM and overcome IFP. For instance, smaller, neutrally charged ZNPs with deformable shapes might exhibit better penetration.

The EPR (enhanced permeability and retention) effect, characterised by leaky tumour vasculature and impaired lymphatic drainage, leads to preferential accumulation of nanoparticles in tumours. However, the extent of the EPR effect varies significantly between patients and even within different tumours in the same patient. ZNP size can be precisely controlled during synthesis. For tumours with smaller pore sizes in their vasculature, smaller ZNPs can be synthesised. Conversely, larger, but still EPR‐effective, ZNPs might be suitable for tumours with more permeable vasculature. The surface charge of ZNPs influences their interaction with blood components and cellular uptake. Tailoring the surface charge (e.g., slightly negative or neutral) can optimise circulation time and minimise non‐specific interactions in a patient‐specific manner. While more complex, ZNP shape can also influence their biodistribution and cellular uptake. Different shapes might exhibit varying degrees of extravasation and cellular internalisation depending on the tumour microenvironment of an individual patient. Pre‐treatment imaging or analysis of tumour vascularity in a patient can guide the selection or synthesis of ZNPs with optimal size and surface properties for enhanced tumour accumulation. The microenvironment at the disease site (e.g., pH, enzyme concentration, redox potential) can vary significantly between patients and even within different regions of a diseased tissue. ZNPs can be engineered to respond to these specific cues, triggering drug release. ZNPs can be designed to release their drug payload in response to the acidic pH often found in tumour microenvironments or intracellular compartments like lysosomes. This can be achieved by incorporating pH‐sensitive polymers or linkers in the ZNP matrix or surface. ZNPs can be modified with substrates that are specifically cleaved by enzymes overexpressed at the disease site (e.g., matrix metalloproteinases in tumours, specific proteases in inflammatory regions). This cleavage leads to drug release. Differences in redox potential between healthy and diseased tissues (e.g., higher reducing environment in tumours) can be exploited by incorporating redox‐sensitive linkers in the ZNP structure, leading to site‐specific drug release. Characterisation of the specific microenvironmental conditions at a patient's disease site can inform the design of ZNPs with tailored release mechanisms for optimal drug activation and efficacy at the intended location.

Integrating imaging agents with ZNP‐based therapeutics allows for real‐time monitoring of drug delivery, biodistribution and therapeutic response in individual patients. This theragnostic approach enables personalised treatment adjustments. ZNPs can be loaded or surface‐modified with various imaging agents, such as fluorescent dyes, MRI contrast agents (e.g., gadolinium chelates, iron oxide nanoparticles) or PET/SPECT radionuclides. Pre‐treatment imaging can identify target sites and guide ZNP design. During treatment, imaging can track ZNP accumulation and drug release. Post‐treatment imaging can assess therapeutic efficacy. For a patient undergoing ZNP‐based chemotherapy, MRI‐tagged ZNPs can be used to monitor drug accumulation in the tumour. If accumulation is insufficient, treatment parameters (e.g., dosage, administration route) can be adjusted in real time. Fluorescently labelled ZNPs can be used in ex vivo diagnostics on patient tissue samples to assess drug penetration and cellular uptake.

Individual patients exhibit varying immune responses to nanoparticles. ZNPs can be modified to minimise adverse immune reactions and potentially modulate the immune system for therapeutic benefit. Coating ZNPs with hydrophilic polymers like PEG (polyethylene glycol) can create a steric barrier that minimises protein adsorption (opsonization) and reduces recognition by the reticuloendothelial system (RES), prolonging circulation time and potentially reducing immunogenicity in specific patients who exhibit rapid clearance of uncoated nanoparticles. ZNPs can be loaded with immunomodulatory drugs or antigens to specifically modulate the immune response in individual patients with autoimmune diseases or for personalised cancer immunotherapy. Patients with a history of hypersensitivity reactions to certain nanoparticles might benefit from ZNPs coated with biocompatible and non‐immunogenic polymers. In cancer immunotherapy, ZNPs can deliver patient‐specific tumour antigens along with adjuvants to stimulate a personalised anti‐tumour immune response.

Individual patients may require unique combinations of therapeutic agents based on their specific disease subtype, genetic profile, or resistance patterns. ZNPs can be used to co‐encapsulate multiple drugs. The inherent structure of ZNPs allows for the encapsulation of both hydrophobic and hydrophilic drugs simultaneously or sequentially during the formulation process. The ratio of different drugs encapsulated within the ZNPs can be precisely controlled to match the personalised therapeutic regimen for a specific patient. For a cancer patient with known drug resistance mutations, ZNPs can be loaded with a combination of drugs that synergistically overcome this resistance, with the drug ratio tailored to the patient's specific resistance profile. In patients with complex conditions requiring multiple medications, ZNPs can co‐deliver these drugs at the target site, potentially improving efficacy and reducing systemic side effects. Patients often exhibit unique expression patterns of specific biomarkers (e.g., overexpressed receptors on cancer cells, specific antigens in inflammatory tissues). ZNPs can be surface‐engineered to target these biomarkers. Targeting ligands such as antibodies, peptides (e.g., RGD for integrins on tumour vasculature), aptamers or small molecules with high affinity for specific biomarkers can be covalently or non‐covalently attached to the ZNP surface. Based on a patient's diagnostic profile (e.g., immunohistochemistry, genetic testing), ZNPs can be functionalised with ligands that specifically recognise the overexpressed biomarkers in their disease. For a patient with a tumour overexpressing EGFR, ZNPs can be conjugated with EGFR‐targeting antibodies or ligands to selectively deliver anticancer drugs to the tumour cells, sparing healthy tissues. In patients with rheumatoid arthritis exhibiting high levels of specific inflammatory markers, ZNPs can be functionalised with ligands that bind to these markers, delivering anti‐inflammatory drugs directly to the inflamed joints.

Polymorphisms in drug‐metabolising enzymes (e.g., CYP450 family) can lead to inter‐individual differences in drug pharmacokinetics. ZNPs can be designed to bypass or overcome these metabolic variations. For example, encapsulating drugs within ZNPs can protect them from enzymatic degradation, potentially leading to more consistent drug levels regardless of a patient's metabolic profile. Polymorphisms in drug target genes can alter the structure or expression level of the target protein, affecting drug binding affinity and efficacy. ZNPs can be functionalised with targeting ligands that have high affinity for the specific polymorphic variant of the target receptor present in a patient's tumour. Genetic variations in immune‐related genes (e.g., HLA genes, cytokine genes) can influence a patient's immune response to both the drug and the nanoparticle itself. ZNP surface modifications (e.g., PEGylation) can be tailored to minimise immunogenicity in patients with specific immune profiles. In cancer immunotherapy, ZNPs can be designed to deliver specific antigens or adjuvants that are most effective based on a patient's HLA type and other immunogenetic markers. Variations in drug transporter genes can affect drug uptake and efflux in target cells. ZNPs can be engineered to utilise specific transporters that are highly expressed or functional in a patient's cells, or to bypass transporters associated with drug resistance due to polymorphisms.

A major hurdle for any nanoparticle‐based therapy is avoiding clearance by the body's immune system before it can reach the tumour. To address this, zein nanoparticles are often coated with PEG in a process known as PEGylation [101]. This PEG coating creates a stealth effect, making the nanoparticles less recognisable to immune cells and increasing their circulation time in the bloodstream [102]. This extended circulation allows for greater accumulation of the nanoparticles at the tumour site through the EPR effect. PEGylation not only improves the stability and longevity of the zein nanoparticles in the body but also enhances their overall therapeutic efficacy.

Besides PEGylation, lipidation and polypeptide‐based modifications like PASylation can also be crucial for enhancing zein‐based nanoparticles in cancer therapy. These modifications can also address limitations such as short half‐lives by increasing hydrodynamic volume and minimising RES uptake, thus prolonging circulation and improving their accumulation in tumour tissues. Lipidation enhances stability and biocompatibility, while PASylation, using naturally derived, non‐immunogenic proline‐alanine‐serine (PAS) sequences, offers a biocompatible stealth coating, which can be genetically encoded. All these modifications can make Zein‐based nanoparticles suitable for cancer therapy by overcoming systemic problems.

ZNPs can be engineered to simultaneously deliver multiple therapeutic agents, such as two different chemotherapeutic drugs, a chemotherapeutic agent and a gene therapy vector (e.g., siRNA to silence drug resistance genes), or a drug and an imaging agent. This co‐delivery can lead to synergistic effects, overcoming drug resistance mechanisms, and enabling real‐time monitoring of drug delivery and therapeutic response. MDR is a major obstacle in cancer treatment. ZNPs can co‐deliver chemotherapeutic drugs with P‐glycoprotein inhibitors or siRNA targeting MDR‐related genes, effectively reversing resistance and improving treatment outcomes. Co‐delivery systems can simplify complex treatment regimens, improve patient compliance, and ensure that multiple drugs reach the target site at the same time and in optimal ratios. Combining ZNP‐based drug delivery with traditional chemotherapy can enhance the efficacy and reduce the toxicity of chemotherapeutic agents. ZNPs can improve drug solubility, prolong circulation time, selectively deliver drugs to tumour sites (via passive or active targeting), and control drug release, thereby maximising the therapeutic index. ZNPs can be utilised to deliver immunotherapeutic agents such as antigens, adjuvants, immune checkpoint inhibitors, or cytokines to the tumour microenvironment or immune cells. This targeted delivery can enhance the anti‐tumour immune response, overcome immunosuppressive mechanisms within the tumour, and improve the overall efficacy of immunotherapy. ZNPs can be designed to co‐deliver both chemotherapeutic drugs and immunotherapeutic agents, creating a synergistic effect that directly kills cancer cells while simultaneously stimulating the immune system to attack the remaining tumour cells and prevent metastasis. Taking advantage of the acidic microenvironment of tumours and intracellular compartments (e.g., lysosomes, endosomes), ZNPs can be engineered to release their drug payload specifically at these sites. This can be achieved by–(a) incorporating pH‐sensitive polymers that undergo conformational changes or degradation at specific pH values, (b) using acid‐labile linkers that cleave in acidic environments, releasing the drug and (c) designing ZNPs with surface charges that change at specific pH, enhancing cellular uptake or disrupting nanoparticle stability. Besides pH, ZNPs can be designed to respond to a variety of other stimuli present in the disease microenvironment or triggered externally. ZNPs can be modified with substrates that are cleaved by enzymes overexpressed in tumours (e.g., MMPs, proteases), leading to localised drug release. ZNPs can be designed to release drugs in response to the higher reducing environment found in tumour cells or the TME. ZNPs incorporating thermo‐responsive polymers can release their payload upon local hyperthermia, which can be induced externally (e.g., using lasers or magnetic fields). ZNPs can be loaded with photo‐responsive molecules that trigger drug release upon irradiation with specific wavelengths of light, allowing for precise spatial and temporal control of drug delivery. Magnetic nanoparticles can be incorporated into or conjugated with ZNPs, allowing for targeted delivery to a specific site using an external magnetic field and potentially triggering drug release via magnetic hyperthermia.

A significant challenge in cancer treatment is the development of chemoresistance. To overcome this, researchers are loading zein nanoparticles with a combination of two or more therapeutic agents. This dual‐drug delivery approach allows for a synergistic attack on cancer cells, targeting multiple cellular pathways simultaneously. For example, a single zein nanoparticle can carry both a conventional chemotherapy drug and a newer, optimised and targeted therapy agent [103]. This combination can be more potent than either drug administered alone, potentially leading to lower required doses and reduced side effects [104]. The hydrophobic nature of zein makes it particularly suitable for encapsulating and delivering water‐insoluble cancer drugs, which are often difficult to formulate [105]. To ensure that the potent anti‐cancer drugs are released only at the tumour site, minimising damage to healthy tissues, zein nanoparticles are being engineered to be stimuli‐responsive [31]. This means they are designed to release their payload in response to specific triggers present in the tumour microenvironment [106]. One of the most common triggers is the slightly acidic pH of tumours compared to normal tissues. As mentioned earlier, by modifying the surface of the zein nanoparticles with pH‐sensitive polymers (e.g., alginate, chitosan), researchers can create a system where the nanoparticle remains stable in the bloodstream but breaks down and releases its drug cargo upon reaching the acidic tumour microenvironment. Other stimuli being explored include specific enzymes that are overexpressed in tumours, externally applied triggers like light or ultrasound or internal conditions such as hypoxia. The elements incorporated into the zein nanoparticle structure can undergo a conformational change, disassemble or trigger drug release only when exposed to these unique TME conditions. This engineering allows the nanoparticles to respond dynamically to the pathological environment of the tumour, making the drug delivery process highly localised and efficient. Furthermore, zein nanoparticles can be designed to deliver agents that reprogram the TME itself, by modulating immunosuppressive cells or disrupting the extracellular matrix, thereby strengthening the overall anti‐tumour response and improving drug penetration. This smart release mechanism enhances the precision of the therapy, concentrating the drug's effect where it is needed most [107].

Artificial intelligence (AI) algorithms can analyse vast datasets of ZNP properties (size, shape, surface charge, composition) and their interactions with biological systems (protein corona formation, cellular uptake, biodistribution) to predict optimal ZNP characteristics for specific applications and patient profiles. Computational models can integrate patient‐specific physiological parameters (e.g., blood flow, organ volumes, metabolic rates) and drug properties with ZNP characteristics to predict drug release kinetics, biodistribution, and therapeutic efficacy in individual patients. This can help optimise dosage and administration schedules. AI can be used to predict potential toxicity based on ZNP properties and patient genetic information, aiding in the selection of safe and biocompatible formulations. Machine learning algorithms can analyse experimental data from ZNP synthesis and formulation studies to identify the optimal process parameters (e.g., zein concentration, solvent ratios, temperature, sonication time) for achieving desired nanoparticle properties (size, encapsulation efficiency and stability) tailored to specific patient needs or drug payloads. AI can analyse large datasets from high‐throughput screening of different ZNP formulations and surface modifications to identify those with the best targeting ability, drug release profiles and cellular uptake in patient‐derived cells or tumouroids. AI can analyse patient data (genomics, proteomics and imaging) to identify subgroups of patients who are most likely to benefit from specific ZNP‐based therapies. Computational models can aid in the design of ZNPs that integrate both therapeutic and imaging functionalities, optimised for individual patient characteristics to enable personalised monitoring of drug delivery and treatment response. AI can predict synergistic drug combinations that can be co‐loaded into ZNPs for personalised therapy based on a patient's specific disease profile and resistance mechanisms.

## 29 Conclusion

The dynamic synergy between cutting‐edge nanotechnology and innovative cancer treatment strategies has ushered in a new era of potential in the field of oncology. The loading of zein nanoparticles with various therapeutic agents (inhibitors, phytocompounds and drugs) and fusion with anticancer peptides or proteins to form supramolecular assemblies, holds exceptional promise as a formidable weapon against the formidable adversary that is cancer. This comprehensive review has highlighted the revolutionary potential of zein nanoparticles as a paradigm for cancer drug delivery. The advancements in nanotechnology have paved the way for improved drug delivery mechanisms, addressing challenges like localisation, targeted delivery and controlled release in cancer treatment. The versatility and unique attributes of zein nanoparticles offer a promising avenue for enhancing the effectiveness of cancer therapeutics. The review delved into the structure, properties and synthesis of zein nanoparticles, showcasing their distinct characteristics such as hydrophobicity, biocompatibility and biodegradability. The variations in synthesis methodologies, including pH‐controlled nanoprecipitation and modification techniques, demonstrate the adaptability of zein nanoparticles to encapsulate a diverse range of therapeutic agents, both hydrophobic and hydrophilic. This adaptability has significant implications for drug delivery, enabling the encapsulation of various compounds to address different cancer types.

The novelty of this review lies in its thorough exploration of ligand‐based modifications of zein nanoparticles for cancer drug delivery. The integration of ligands for site‐specific targeting presents an innovative approach to enhancing drug delivery accuracy and effectiveness. The incorporation of ligands not only improves targeting but also offers potential solutions to drug resistance in cancer therapies. Moreover, the discussion on gene therapy using zein nanoparticles for cancer treatment underscores the broader scope of their application. The ability to encapsulate genes and deliver them to target cells opens new doors in the field of personalised medicine and precise therapeutic interventions. Examples of gene therapy strategies utilising zein nanoparticles, such as PTEN and TRAIL loaded zein nanoparticles for hepatocellular carcinoma treatment, demonstrate the potential for gene‐specific targeting and modulation. The presented studies on various anticancer agents, such as curcumin, doxorubicin, paclitaxel, and more, encapsulated within zein nanoparticles, underscore the versatility of this delivery system. The results of in vitro and in vivo experiments emphasise the enhanced cytotoxicity, apoptosis induction, and anti‐migratory effects of these formulations, highlighting their potential to revolutionise cancer treatment outcomes.

## Author Contributions

**Hanan M. Alharbi:** conceptualization (equal), writing – original draft (equal), writing – review and editing (equal). **Taha Alqahtani:** conceptualization (equal), validation (equal), writing – review and editing (equal). **Nada A. Alqalawi:** conceptualization (equal), validation (equal), writing – original draft (equal). **Shayma A. Alsayegh:** conceptualization (equal), validation (equal), writing – original draft (equal). **Basmah A. Almaghrabi:** conceptualization (equal), validation (equal), writing – review and editing (equal). **Subham Sarkar:** conceptualization (equal), software (equal), writing – original draft (equal). **Daniel Ejim Uti:** conceptualization (equal), writing – original draft (equal), writing – review and editing (equal). **Bikram Dhara:** conceptualization (equal), validation (equal), writing – review and editing (equal).

## Conflicts of Interest

The authors declare no conflicts of interest.

## Data Availability

All the data used are available within the manuscript.
